# Activation of the pentose phosphate pathway in macrophages is crucial for granuloma formation in sarcoidosis

**DOI:** 10.1172/JCI171088

**Published:** 2023-12-01

**Authors:** Satoshi Nakamizo, Yuki Sugiura, Yoshihiro Ishida, Yoko Ueki, Satoru Yonekura, Hideaki Tanizaki, Hiroshi Date, Akihiko Yoshizawa, Teruasa Murata, Kenji Minatoya, Mikako Katagiri, Seitaro Nomura, Issei Komuro, Seishi Ogawa, Saeko Nakajima, Naotomo Kambe, Gyohei Egawa, Kenji Kabashima

**Affiliations:** 1 Department of Dermatology,; 2 Alliance Laboratory for Advanced Medical Research, and; 3 Center for Cancer Immunotherapy and Immunobiology, Kyoto University Graduate School of Medicine, Kyoto, Japan.; 4 Department of Dermatology, Kansai Medical University, Osaka, Japan.; 5 Department of Thoracic Surgery and; 6 Department of Diagnostic Pathology, Kyoto University Graduate School of Medicine, Kyoto, Japan.; 7 Department of Dermatology, Hyogo College of Medicine, Nishinomiya, Japan.; 8 Department of Cardiovascular Surgery, Kyoto University Graduate School of Medicine, Kyoto, Japan.; 9 Department of Cardiovascular Medicine and; 10 Department of Frontier Cardiovascular Science, Graduate School of Medicine, The University of Tokyo, Tokyo.; 11 International University of Health and Welfare, Tokyo, Japan.; 12 Department of Pathology and Tumor Biology, Graduate School of Medicine, Kyoto University, Kyoto, Japan.; 13 Institute for the Advanced Study of Human Biology (WPI-ASHBi), Kyoto University, Kyoto, Japan.; 14 Department of Drug Discovery for Inflammatory Skin Diseases, Kyoto University Graduate School of Medicine, Kyoto, Japan.; 15 Skin Research Institute of Singapore (SRIS) and A*STAR Skin Research Labs (A*SRL), Agency for Science, Technology, and Research (A*STAR), Singapore.

**Keywords:** Dermatology, Immunology, Antigen-presenting cells, Macrophages, Skin

## Abstract

Sarcoidosis is a disease of unknown etiology in which granulomas form throughout the body and is typically treated with glucocorticoids, but there are no approved steroid-sparing alternatives. Here, we investigated the mechanism of granuloma formation using single-cell RNA-Seq in sarcoidosis patients. We observed that the percentages of triggering receptor expressed on myeloid cells 2–positive (TREM2-positive) macrophages expressing angiotensin-converting enzyme (ACE) and lysozyme, diagnostic makers of sarcoidosis, were increased in cutaneous sarcoidosis granulomas. Macrophages in the sarcoidosis lesion were hypermetabolic, especially in the pentose phosphate pathway (PPP). Expression of the PPP enzymes, such as fructose-1,6-bisphosphatase 1 (FBP1), was elevated in both systemic granuloma lesions and serum of sarcoidosis patients. Granuloma formation was attenuated by the PPP inhibitors in in vitro giant cell and in vivo murine granuloma models. These results suggest that the PPP may be a promising target for developing therapeutics for sarcoidosis.

## Graphical abstract

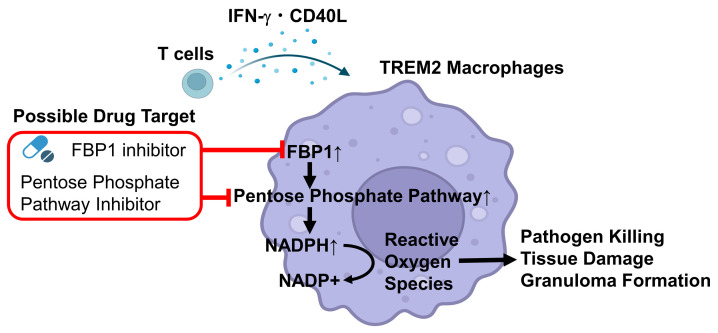

## Introduction

Granulomas are chronic inflammatory lesions having well-defined borders and comprising mainly macrophages and other inflammatory cells (1). Differentiation of macrophages into epithelioid cells and multinucleated giant cells is often observed. Sarcoidosis is a typical granulomatosis disease in which granulomas usually develop in organs throughout the body, including the skin, lungs, lymph nodes, heart, and eyes (2). Sarcoidosis may resolve within a few years without treatment, but can persist for decades (3). In severe cases, sarcoidosis can lead to pulmonary fibrosis, blindness, and heart failure. An analysis of the economic impact of sarcoidosis in the United States indicates a significant burden, as evidenced by the total annual health-care costs incurred by commercial payers per patient within the first year after diagnosis, which is approximately $19,714 (4). In addition to symptomatic treatment with steroids, treatment with TNF-α–neutralizing antibodies and Janus kinase inhibitors has been considered for sarcoidosis (2, 5, 6). However, these therapies have significant side effects, such as immunosuppression. Novel therapeutic directions are, therefore, urgently needed.

Uncovering the pathogenesis of sarcoidosis is essential to developing specific therapies. Granuloma typically manifests as a reaction to exogenous foreign bodies that have been left undigested for a long period of time (1). Compared with nonfused macrophages, giant cells generally show enhanced levels of ROS and acid production that together contribute to the rejection of foreign substances and microorganisms (7, 8). ROS generated by NADPH oxidase (NOX), as well as cytokines and many adhesion proteins and receptors, plays an important role in macrophage fusion (9). Disruption of ROS production by causative variants in the NOX gene renders patients highly susceptible to infection, underscoring the importance of ROS production in innate immunity (10). Whole genome-sequencing results indicated that genes associated with NOX may be involved in the etiology of sarcoidosis (11).

Variations in metabolic processes, including glucose uptake and metabolism, mitochondrial function, amino acid uptake, and cholesterol and lipid synthesis in macrophages, are correlated with alterations in cytokine production and phagocytic activity (12). One example is the upregulation of the pentose phosphate pathway (PPP), which is crucial for maintaining the NADPH supply during acute oxidative stress. Lipopolysaccharide-stimulated macrophages display an elevated production of ROS through NADPH and NOX by activating the PPP (13). However, the mechanism behind the generation of NADPH in granulation-forming macrophages remains poorly understood.

Here, to identify regulators of sarcoidosis granuloma formation, we have employed single-cell RNA-sequencing (scRNA-Seq) to examine cutaneous sarcoidosis samples. Our findings showed that only triggering receptors expressed on myeloid cells 2–positive (TREM2-positive) macrophages (TREM2 macrophages) expressing angiotensin-converting enzyme (ACE) and lysozyme, diagnostic markers of sarcoidosis, were found to be increased in cutaneous sarcoidosis granulomas. Of note, these TREM2 macrophages were in a hypermetabolic state, with increased expression of fructose-1,6-bisphosphatase 1 (FBP1) and other enzymes involved in metabolism. Consistently, FBP1-positive TREM2 macrophages were also found in the granulomas in the lung, heart, and lymph nodes of sarcoidosis patients. Elevated serum FBP1 levels were also observed in sarcoidosis patients. A subset of FBP1-positive TREM2 macrophages expressing genes involved in cell fusion also had elevated PPP-related genes linked to ROS production. Inhibiting the PPP resulted in suppression of giant cell formation in vitro and granuloma formation in a murine granuloma model, demonstrating that inhibiting PPP may be a potential therapeutic approach for treating granulomatous diseases, such as sarcoidosis, recalcitrant to current options.

## Results

### FBP1-positive TREM2 macrophages constitute granulomas in sarcoidosis.

So that we could understand the mechanisms regulating granuloma formation in sarcoidosis, scRNA-Seq was performed on lesional skin samples from 3 patients with sarcoidosis and 5 healthy subjects (Supplemental Table 1; supplemental material available online with this article; https://doi.org/10.1172/JCI171088DS1). Unsupervised clustering revealed 10 major cell subsets in all 8 samples: fibroblasts, keratinocytes, T cells, vascular endothelial cells, pericytes and vascular smooth muscle cells, antigen-presenting cells (APCs), lymphatic endothelial cells, melanocytes, nerve cells, and proliferating cells (Supplemental Figure 1, A–E). Cell identities were annotated using cell subset–specific markers.

**Figure 1:**
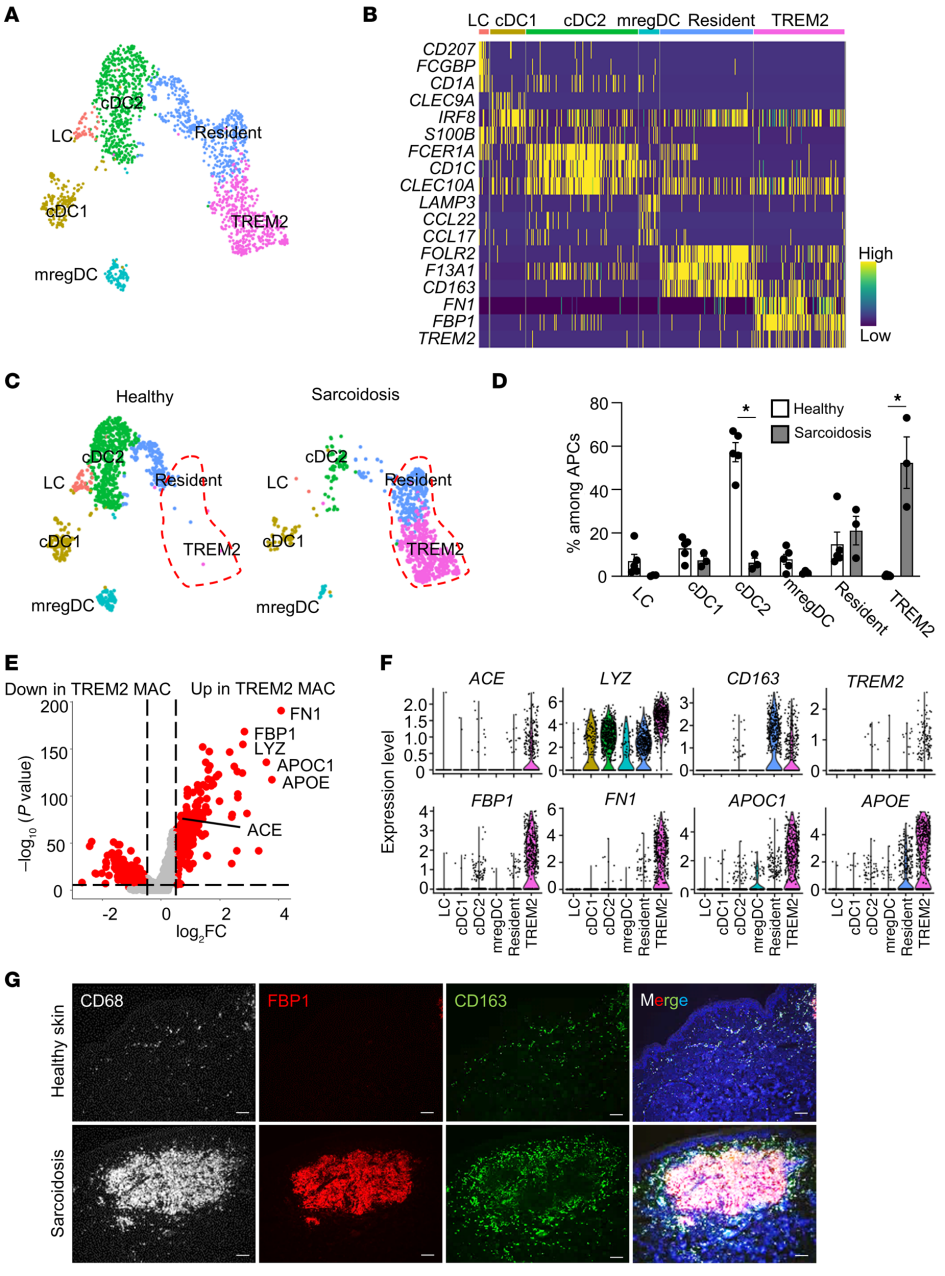
Identification of APC subsets. (**A**) UMAP plot showing APCs from 3 sarcoidosis patients and 5 healthy subjects colored by subset. LC, Langerhans cells; cDC1, conventional type 1 dendritic cells; cDC2, conventional type 2 dendritic cells; mregDC, mature dendritic cells enriched in immunoregulatory molecules; resident, skin resident macrophages; TREM2, TREM2 macrophages. (**B**) Heatmap showing marker genes for each subset. Representative genes are labeled. (**C**) UMAP plot split by healthy and sarcoidosis samples. (**D**) An abundance of each APC subset. Šídák’s multiple-comparisons test was conducted between the healthy and sarcoidosis samples for each subset. *P < 0.05. (**E**) Volcano plot comparing TREM2 macrophages against other APCs. MAC, macrophages. (**F**) Violin plots showing expression of *ACE*, *LYZ*, *CD163*, *TREM2*, *FBP1*, *FN1*, *APOC1,* and *APOE* in APC subsets in all skin samples. (**G**) Representative immunofluorescence staining in healthy (*n* = 3) and sarcoidosis (*n* = 3) skin biopsy samples for expression of CD68 in gray, FBP1 in red, CD163 in green, and DAPI in blue. Scale bars: 100 μm.

Since sarcoidosis is primarily a disease of granuloma-forming macrophages, APCs were the main focus of subsequent analyses. Based on previous reports, cutaneous APCs were subdivided into CD207-positive Langerhans cells, CLEC9A-positive conventional type 1 dendritic cells, CD1C-positive conventional type 2 dendritic cells, LAMP3-positive mature dendritic cells enriched in immunoregulatory molecules, CD163-positive skin-resident macrophages (resident macrophages), and TREM2-positive macrophages (TREM2 macrophages) (Figure 1, A and B) (14). TREM2 macrophages, rarely present in healthy skin, represented half of the APCs and a decreased percentage of conventional type 2 dendritic cells in sarcoidosis skin (Figure 1, C and D). Immunostaining revealed that both the overall count of APCs and the specific numbers of conventional type 2 dendritic cells and macrophages were elevated in sarcoidosis skin (Supplemental Figure 2A). In sarcoidosis granuloma samples, the expression of genes such as *ACE* and lysozyme (*LYZ*) is known to be elevated, and these genes were indeed upregulated in TREM2 macrophages (Figure 1, E and F). Of note, TREM2 macrophages showed elevated expression of fibronectin (*FN1*), *FBP1*, apolipoprotein C1 (*APOC1*), and *APOE*, which are related to cell adhesion and glucose and lipid metabolism. Given that expression of FBP1 was restricted to macrophages, whereas FN1 and APOE were also present on fibroblasts, lymphatic endothelial cells, and melanocytes, we opted to utilize FBP1 as a marker for TREM2 macrophages (Supplemental Figure 1F). Immunohistochemistry of sarcoidosis skin samples showed positivity for TREM2 and FBP1, which is consistent with the accumulation of CD68-positive cells in granuloma areas (Figure 1G and Supplemental Figure 2, B–D). The granulomas were surrounded by cells positive for CD163, a resident macrophage subset marker. In contrast, no FBP1-positive cells were observed in healthy skin. These findings indicate that FBP1-positive TREM2 macrophages make up sarcoidosis granulomas.

### FBP1 is expressed in macrophages associated with cutaneous and noncutaneous granulomatous diseases.

**Figure 2:**
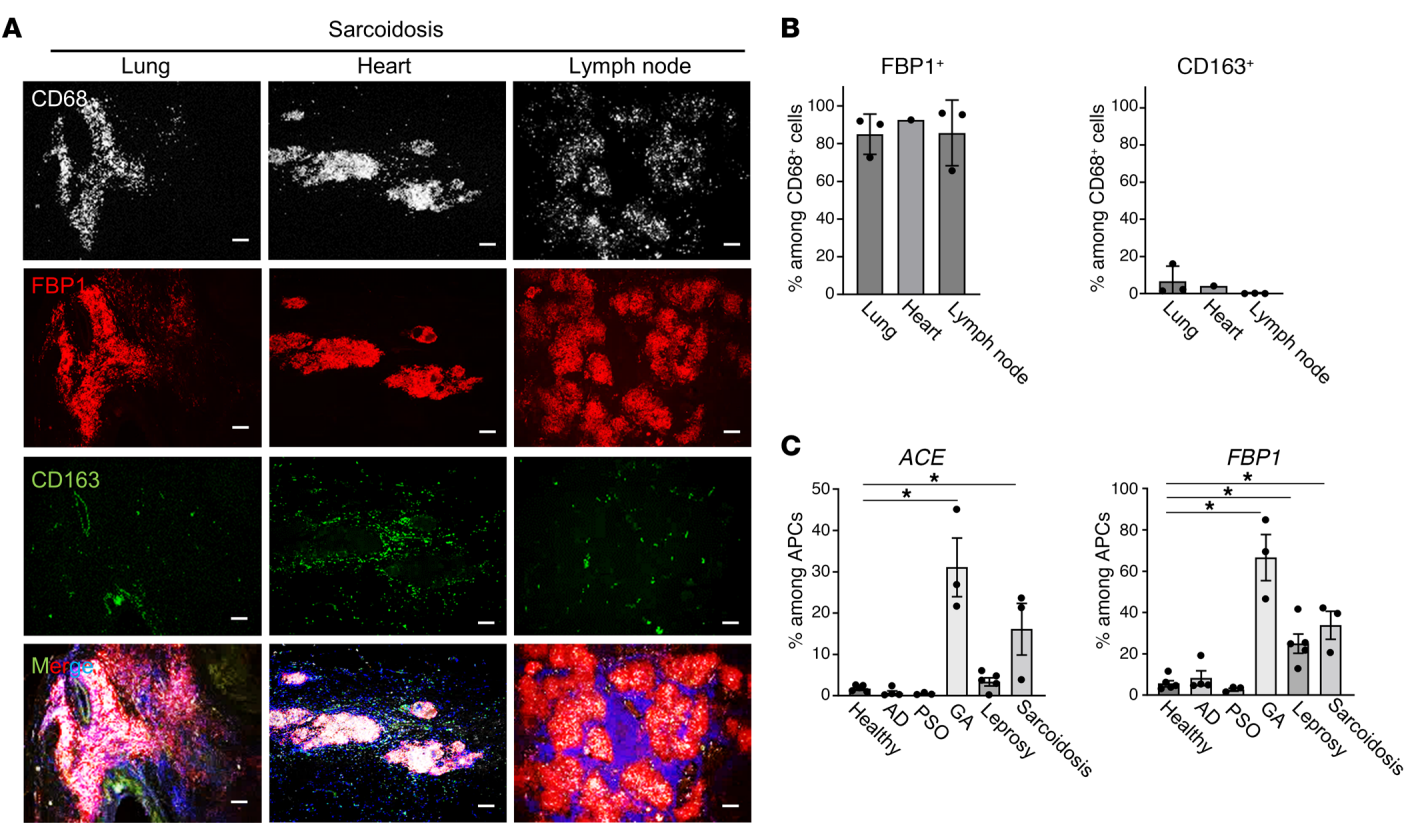
FBP1 is expressed in macrophages in association with cutaneous and noncutaneous sarcoidosis. (**A** and **B**) Representative immunofluorescence staining (**A**) and bar graph (**B**) in extradermal lesions (lung, *n* = 3; heart, *n* = 1; lymph node, *n* = 3) of sarcoidosis samples for expression of CD68 in gray, FBP1 in red, CD163 in green, and DAPI in blue. Scale bars: 100 μm. (**C**) Frequency of ACE^+^ and FBP1^+^ APCs in inflammatory skin diseases based on previously published data (16–18). AD, atopic dermatitis; PSO, psoriasis; GA, granuloma annulare. *P < 0.05, Dunnett’s multiple-comparisons test.

Since sarcoidosis results in granulomas in various organs throughout the body, we examined FBP1 expression in granulomas of tissues other than the skin by immunostaining. FBP1 expression was also detected in granulomas of the lung, heart, and lymph nodes (Figure 2, A and B). FBP1 expression was detected in CD68-positive areas, while CD163 was observed surrounding FBP1-positive areas in all organs. These results indicate that in sarcoidosis, CD68-positive macrophages within the granuloma express FBP1, regardless of the organ.

We also compared gene expression in FBP1-positive TREM2 macrophages in the skin and heart using single-nucleus RNA-Seq data for cardiac sarcoidosis in public databases (15). Genes highly expressed in cardiac FBP1-positive TREM2 macrophages were also highly expressed in skin FBP1-positive TREM2 macrophages (Supplemental Figure 3A). Thus, FBP1-positive TREM2 macrophages share a typical phenotype among organs involved in sarcoidosis.

**Figure 3:**
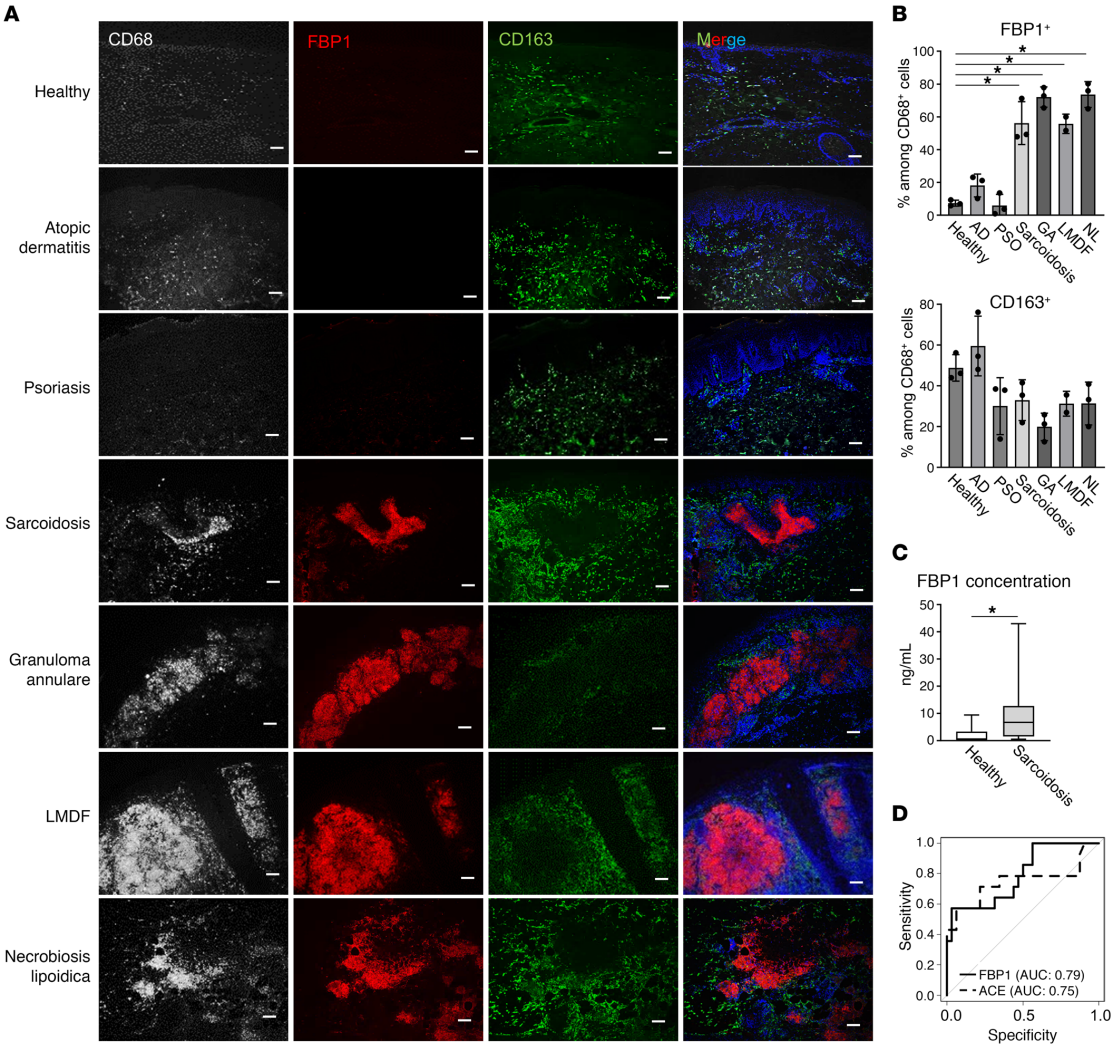
FBP1 is expressed in macrophages in association with cutaneous granulomatous diseases. (**A** and **B**) Representative immunofluorescence staining (**A**) and bar graph (**B**) in healthy and inflammatory skin disease samples (*n* = 3) for expression of CD68 in gray, FBP1 in red, CD163 in green, and DAPI in blue. Scale bars: 100 μm. *P < 0.05, Dunnett’s multiple-comparisons test. LMDF, lupus miliaris disseminatus faciei. (**C** and **D**) Box-and-whisker plots (**C**) and receiver operating characteristic curves (**D**) of serum FBP1 and ACE concentrations in healthy subjects (*n* = 32) and sarcoidosis patients (*n* = 14). Mann-Whitney *U* tests were conducted between healthy and sarcoidosis samples. *P < 0.05.

The proportions of APCs expressing ACE (a previously established diagnostic marker for sarcoidosis) and FBP1 (a newly discovered marker in this study) in skin lesions were analyzed in granulomatous skin diseases other than sarcoidosis. In addition, data from previously published databases for nongranulomatous inflammatory skin diseases, such as atopic dermatitis and psoriasis (16), and granulomatous skin diseases, such as granuloma annulare, leprosy, and sarcoidosis, were interrogated (17, 18). APCs expressing ACE and FBP1 were detected in all granulomatous diseases examined (Figure 2C). To evaluate the universality of FBP1-positive TREM2 macrophages across granulomatous skin diseases, we performed immunostaining on healthy skin as well as samples from patients with inflammatory and granulomatous skin diseases. In granulomatous skin disease, FBP1 expression was detected in CD68-positive granuloma regions, while CD163 was observed surrounding FBP1-positive regions (Figure 3, A and B). In contrast, no FBP1-positive cells were observed in nongranulomatous inflammatory skin diseases. These results indicate that FBP1 is expressed in CD68-positive macrophages within the granulomas in cutaneous and noncutaneous granulomatous diseases, but not in inflammatory skin diseases.

### Serum FBP1 levels were elevated in sarcoidosis patients.

To investigate the potential of FBP1 as a biomarker in sarcoidosis, serum FBP1 levels in healthy subjects and patients with sarcoidosis were assayed. Serum concentrations of FBP1 were significantly elevated in patients with sarcoidosis (Figure 3C). In addition, calculation of AUCs for receiver operating characteristic curves of serum ACE and FBP1 indicated that FBP1 has a diagnostic capacity comparable to that of ACE (Figure 3D).

### Metabolically active TREM2 macrophages express genes associated with giant cells.

**Figure 4:**
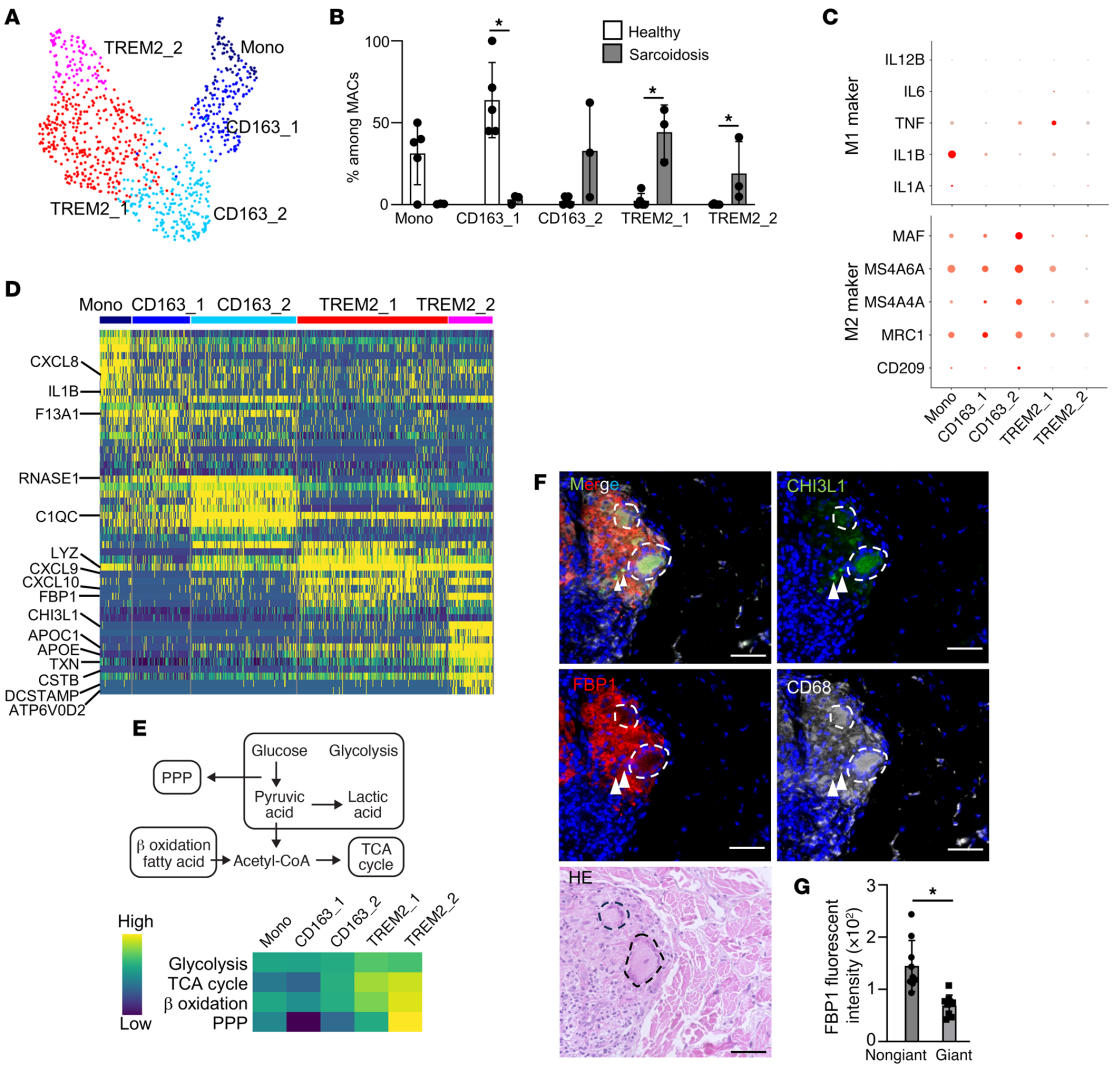
Metabolically active TREM2 macrophages express genes in association with giant cells. (**A**) UMAP plots for all skin scRNA-Seq data (5 healthy subjects and 3 patients) showing macrophage subsets. (**B**) An abundance of each macrophage subset in all skin scRNA-Seq data (5 healthy subjects and 3 patients). Šídák’s multiple-comparisons test was conducted between the healthy and sarcoidosis samples for each subset. *P < 0.05. (**C**) Dot plot of genes upregulated in M1and M2 macrophages in macrophage fractions of scRNA-Seq data (5 healthy subjects and 3 patients). (**D**) Heatmap showing marker genes for each subset in all skin scRNA-Seq data (5 healthy subjects and 3 patients). Representative genes are labeled. (**E**) Heatmap showing metabolic pathway expression for each cell subset in all skin scRNA-Seq data (5 healthy subjects and 3 patients) via Reactome pathway database. (**F** and **G**) Representative H&E and immunofluorescence (**F**) staining and bar graph (**G**) in sarcoidosis skin samples (*n* = 3) for expression of CD68 (gray), FBP1 (red), CHI3L1 (green), and DAPI (blue). Dotted lines indicate giant cells, and arrowheads point to FBP1^+^CHI3L1^+^ cells. Scale bars: 50 μm. Mann-Whitney *U* tests were conducted between nongiant and giant cells. *P < 0.05.

Macrophages that constitute granulomas are heterogeneous and include epithelioid cells and giant cells. Reanalysis of scRNA-Seq data of macrophages revealed that resident macrophages could be classified into monocytes, CD163_1 macrophages or CD163_2 macrophages, and TREM2 macrophages as TREM2_1 or TREM2_2 macrophages (Figure 4A).

Macrophages in healthy skin were composed of monocytes and CD163_1 macrophages, whereas in sarcoidotic skin, macrophages were composed of CD163_2, TREM2_1, and TREM2_2 macrophages (Figure 4B). Monocytes prominently exhibited M1 macrophage markers (19) (Figure 4C). Conversely, CD163 macrophages were associated with the expression of M2 macrophage markers. Monocytes showed high expression of genes relative to inflammatory responses such as *CXCL8* and *IL1B*, whereas CD163_1, and CD163_2 macrophages expressed blood coagulation factors such as coagulation factor XIII A chain (*F13A1*). CD163_2 macrophages express inflammatory genes such as ribonuclease pancreatic (*RNASE1*) and complement C1q C chain (*C1QC*). TREM2_1 and TREM2_2 macrophages showed high expression of genes related to phagocytes including *LYZ* and chemokines such as *CXCL9* and *CXCL10*. TREM2_2 macrophages had high expression of genes relative to metabolism (e.g., *APOC1*, *APOE*) and genes relative to antioxidant production and antiapoptosis, such as thioredoxin (*TXN*) and chitinase-3-like protein 1 (*CHI3L1*), respectively (Figure 4D). TREM2_2 macrophages were also expressed genes that are expressed in osteoclasts, the most studied giant cells, such as cystatin B (*CSTB*) and adenosine triphosphatase H^+^ transporting V0 subunit d2 (*ATP6V0D2*), and dendritic cell–specific transmembrane protein *(DCSTAMP*), which are essential molecules for bone destruction and cell fusion. In addition, using Metascape analysis (https://metascape.org/gp/index.html#/main/step1), we found nuclear transcription factors such as PU.1 (*SPI1*) and *NFKB1*, which promote transcription of DC-STAMP, were demonstrated to be highly expressed in TREM2_2 macrophages (Supplemental Figure 3B). These results suggest that TREM2_2 macrophages may be similar to giant cells. Pathway analysis revealed that TREM2_2 macrophages had enhanced synthesis of ROS and respiratory ATP, identical to that observed in neutrophils (Supplemental Figure 3C).

Since the expression of genes involved in metabolism was elevated in TREM2_2 macrophages, the metabolic pathways activated in each macrophage subset were assessed. Expression of genes related to the TCA cycle, which consumes oxygen to produce ATP, and β-oxidation, which is necessary for fatty acid consumption in the TCA cycle, was upregulated in TREM2_2 macrophages (Figure 4E). Additionally, genes encoding components of the PPP, which generates NADPH, a component of ROS generation, were highly expressed in TREM2_2 macrophages.

The localization of TREM2_2 macrophages in sarcoidosis skin was assessed by immunostaining. CHI3L1, which is highly expressed in TREM2_2 macrophages, was stained in giant cells and cells surrounding giant cells, whereas FBP1 was stained throughout the granuloma except in giant cells (Figure 4, F and G). Granulomas were found to be composed of 3 distinct cellular subsets: FBP1-positive, CHI3L1-negative TREM2_1 macrophages, FBP1- and CHI3L1-expressing TREM2_2 macrophages, and FBP1-negative, CHI3L1-positive giant cells. TREM2_2 macrophages are hypothesized to be implicated in giant cell formation owing to their expression of a plethora of genes associated with cell fusion and metabolism. In contrast, giant cells could not be detected in this scRNA-Seq data set because of their substantial size, which may not be amenable to recovery through the 10X protocol employed in this study.

### The PPP is upregulated in granulomas.

**Figure 5:**
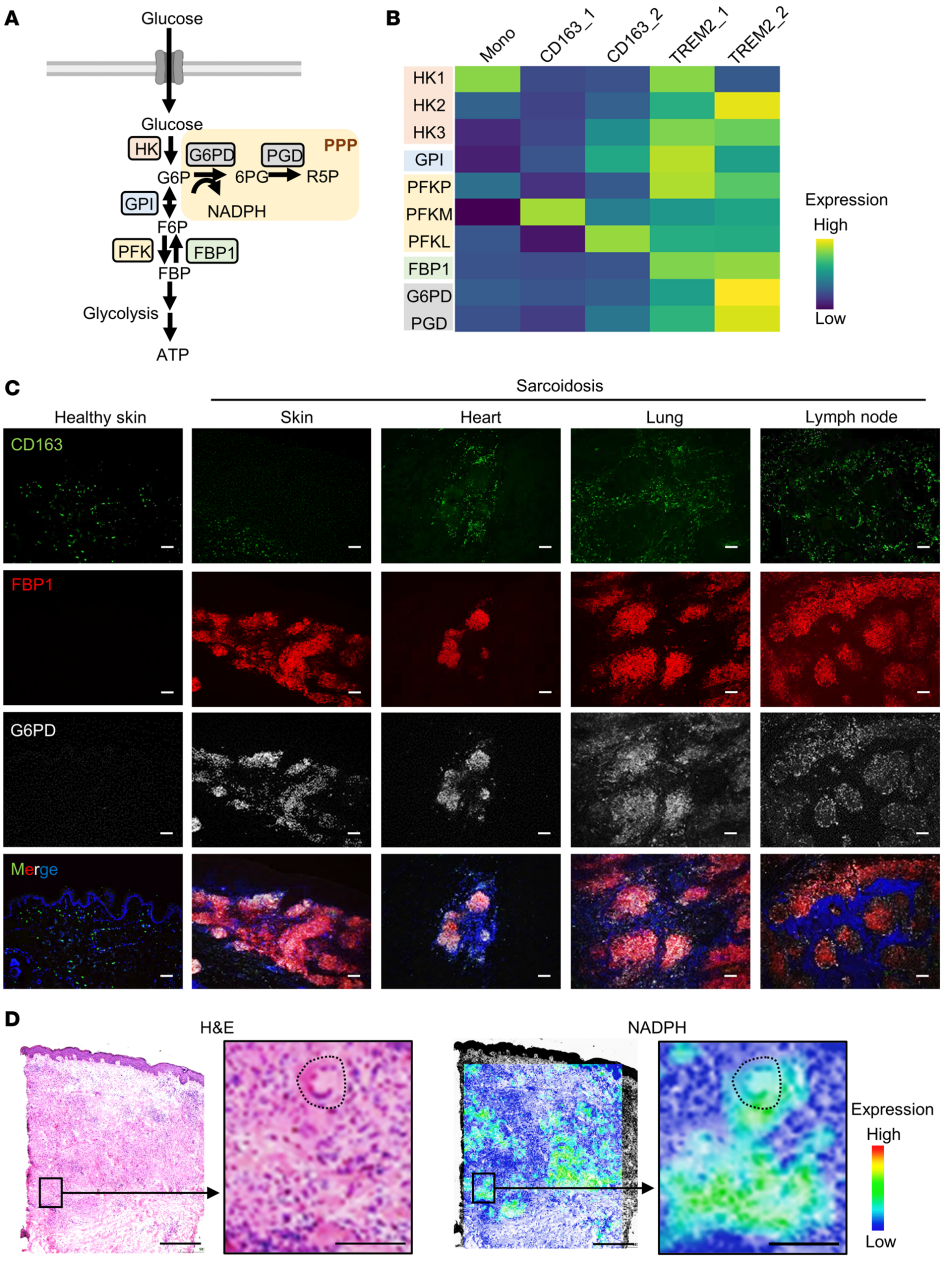
FBP1-positive macrophages switch to PPP as the dominant route for glucose metabolism. (**A**) Schematic diagram of glucose metabolized by the glycolytic system or via the PPP. (**B**) Heatmap showing metabolomic changes for each macrophage subset in all skin scRNA-Seq data (5 healthy subjects and 3 patients). Mono, monocytes. (**C**) Representative immunofluorescence staining in healthy (*n* = 3) and sarcoidosis samples (lung, *n* = 3; heart, *n* = 1; lymph node, *n* = 3) for expression of G6PD in gray, FBP1 in red, CD163 in green, and DAPI in blue. Scale bars: 100 μm. (**D**) H&E-stained sections and imaging for MS analysis of NADPH in the skin of sarcoidosis patients. Dotted lines indicate giant cells Scale bars: 500 μm (left); 100 μm (right).

Granulomas were composed of distinct cell subsets, including TREM2_1, TREM2_2, and giant cells. Each of these cells displayed altered gene expression profiles related to macrophage metabolism, particularly regarding the PPP, such as FBP1. FBP1 increases the number of glucose metabolites entering the PPP by converting FBP back to fructose 6-phosphate (F6P). Glucose-6-phosphate dehydrogenase (G6PD) is the first enzyme that allows glucose metabolites to enter the PPP to produce NADPH (Figure 5A) (20). Expression levels of the PPP enzyme-coding genes, including G6PD and phosphogluconate dehydrogenase (PGD), were increased in TREM2 macrophages, particularly in TREM2_2 macrophages (Figure 5B). Immunostaining showed that FBP1-positive granulomas expressed G6PD regardless of anatomical site of the lesion (Figure 5C and Supplemental Figure 4, A and B). In atopic dermatitis and psoriasis, a G6PD signal was observed at sites of inflammatory cell infiltration. This is most likely because neutrophils express G6PD. No expression of G6PD was observed in healthy skin.

Mass spectrometry (MS) imaging of NADPH production in sarcoidosis granulomas revealed an increase in NADPH consistent with granuloma sites (especially giant cells) in sarcoidosis patients (Figure 5D). These results indicate that the PPP is upregulated in sarcoidosis granulomas. Since NADPH is oxidized by NOX to generate ROS, we examined the expression of NOX in macrophages using scRNA-Seq data and found that NOX2 (*CYBB*) was expressed in macrophages in sarcoidosis skin as previously reported (21, 22) (Supplemental Figure 4C). Expression of Ras-related C3 botulinum toxin substrate 2 (*RAC2*) and neutrophil cytosolic factor 2 (*NCF2* [p67phox]) genes, which interact with NOX and trigger its activation, was upregulated in TREM2_2 macrophages in humans (Supplemental Figure 4D). RAC2 has a 6-fold higher level of binding to p67phox relative to RAC1 and is more efficient at oxidase activation than RAC1 (23). This suggests that NOX activity is enhanced in TREM2_2 macrophages in sarcoidosis skin lesions.

### Inhibition of the PPP impairs the formation of giant cells in an in vitro model.

In sarcoidosis, the production of ROS is enhanced and is thought to contribute to tissue destruction around the granulomas (24). The production of ROS primarily occurs in giant cells; (7, 8) however, this study has also demonstrated an enhancement of ROS in TREM2_2 macrophages. In addition, the expression of genes implicated in cell fusion was markedly elevated in both cell subsets. Therefore, using an in vitro giant cell model, we investigated the correlation between giant cell formation and PPP activity, which is essential for ROS generation.

**Figure 6:**
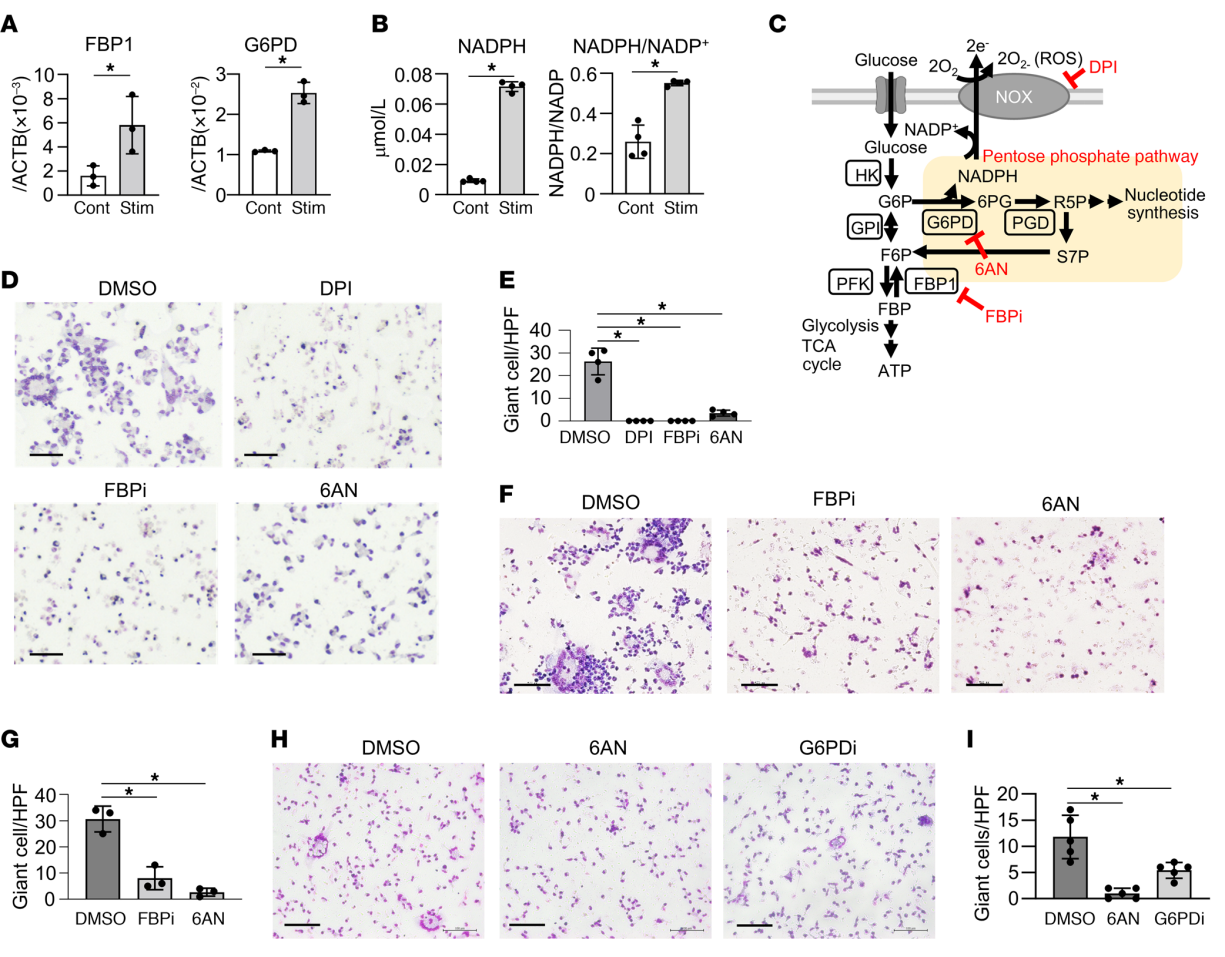
Treatment of in vitro giant cell model by inhibition of the PPP. (**A**) Fold induction of FBP1 and G6PD mRNA in Con A, IFN-γ, and anti-CD40 antibodies stimulated (stim) monocytes, as analyzed by quantitative PCR (*n* = 3). (**B**) Measurement of intracellular NADP and NADPH levels in the in vitro giant cell model (*n* = 4). (**C**) Schematic diagram of metabolic pathways and inhibitors of glucose and NADPH metabolized by the glycolytic system or by the PPP. (**D** and **E**) Giemsa staining (**D**) and bar graphs (**E**) of monocytes stimulated with Con A, IFN-γ, and anti-CD40 antibodies, with or without DMSO, DPI, FBPi, and 6AN (*n* = 4). (**F** and **G**) Giemsa staining (**F**) and giant cell count (**G**) of 3 day cultures stimulated with Con A, IFN-γ, and anti-CD40 antibodies followed by incubation with DMSO, FBPi, or 6AN for 3 days (*n* = 3–5). (**H** and **I**) Giemsa staining (**H**) and bar graphs (**I**) of monocytes stimulated with Con A, IFN-γ, and anti-CD40 antibodies, with or without DMSO, 6AN, or G6PDi (*n* = 5). Data are representative of at least 3 independent experiments. Scale bars: 100 μm. (**A** and **B**) *P < 0.05, unpaired *t* test. (**E**, **G**, and **I**) *P < 0.05, Dunnett’s multiple-comparisons test.

Peripheral blood CD14-positive monocytes from healthy subjects cultured for 3 days with IFN-γ, anti-CD40 antibody, and concanavalin A (Con A) formed Langhans-type giant cells, as reported (25). During giant cell formation, the expression of the PPP-related genes, such as FBP1 and G6PD, increased (Figure 6A). Intracellular NADPH and the NADPH/NADP^+^ ratio were also increased (Figure 6B). These results suggest that the enhancement of NADPH was not due to a change in the number of macrophages, but rather an increase in the amount of NADPH produced within the cells. Thus, our in vitro model of giant cell formation demonstrates an increase in the PPP.

Next, the effect of the PPP inhibitors on giant cell formation was assessed. We added 6-aminonicotinamide (6AN), a G6PD inhibitor, and 2,5-dichloro-N-(5-chloro-2-benzoxazolyl)-benzenesulfonamide, an FBP1 inhibitor (FBPi) in this in vitro granuloma model (Figure 6C). The PPP inhibition with 6AN and FBPi suppressed granuloma formation in an in vitro giant cell model almost as well as direct inhibition of the NOX complex with diphenyleneiodonium chloride (DPI), which reduces ROS production (Figure 6, D and E). The addition of these inhibitors disrupted giant cell formation even after the establishment of giant cell formation (Figure 6, F and G). We also performed experiments using the 4-((5-oxo-6,7,8,9-tetrahydro-5H-cyclohepta[d]pyrimidin-2-yl)amino)thiophene-2-carbonitrile, an G6PD inhibitor (G6PDi) (26). While G6PDi is believed to possess a more specific inhibitory action on G6PD compared with 6AN, our results indicated that G6PDi similarly suppressed giant cell formation in vitro (Figure 6, H and I).

To assess the cytotoxicity resulting from PPP inhibition, we evaluated whether cell mortality increased following the addition of PPP inhibitors in an in vitro giant cell model. Our results indicate that introducing the PPP inhibitor prior to giant cell formation does not increase the percentage of dead cells (Supplemental Figure 5A). In contrast, the addition of the PPP inhibitor after giant cell formation led to an increased percentage of dead cells (Supplemental Figure 5B). This observation aligns with prior research that demonstrated that the inhibition of PPP activity results in cell death, attributed to stimulus-induced oxidative stress (27). We hypothesize that under steady-state conditions, PPP inhibition curtails differentiation into giant cells. However, once the giant cells are formed and encounter heightened oxidative stress, there is an increased propensity for cell death.

To further understand the effect of PPP inhibition on cytokine production in our in vitro granuloma model, we measured cytokine levels in the supernatant. The data revealed elevated expression of IL-1β, IL-6, and TNF-α, all known to reflect the pathogenesis of sarcoidosis (28), in the in vitro granuloma model (Supplemental Figure 5C). However, the application of FBPi or 6AN led to a marked reduction in the production of these cytokines, suggesting that PPP inhibition may concurrently suppress cytokine production in macrophages.

**Figure 7:**
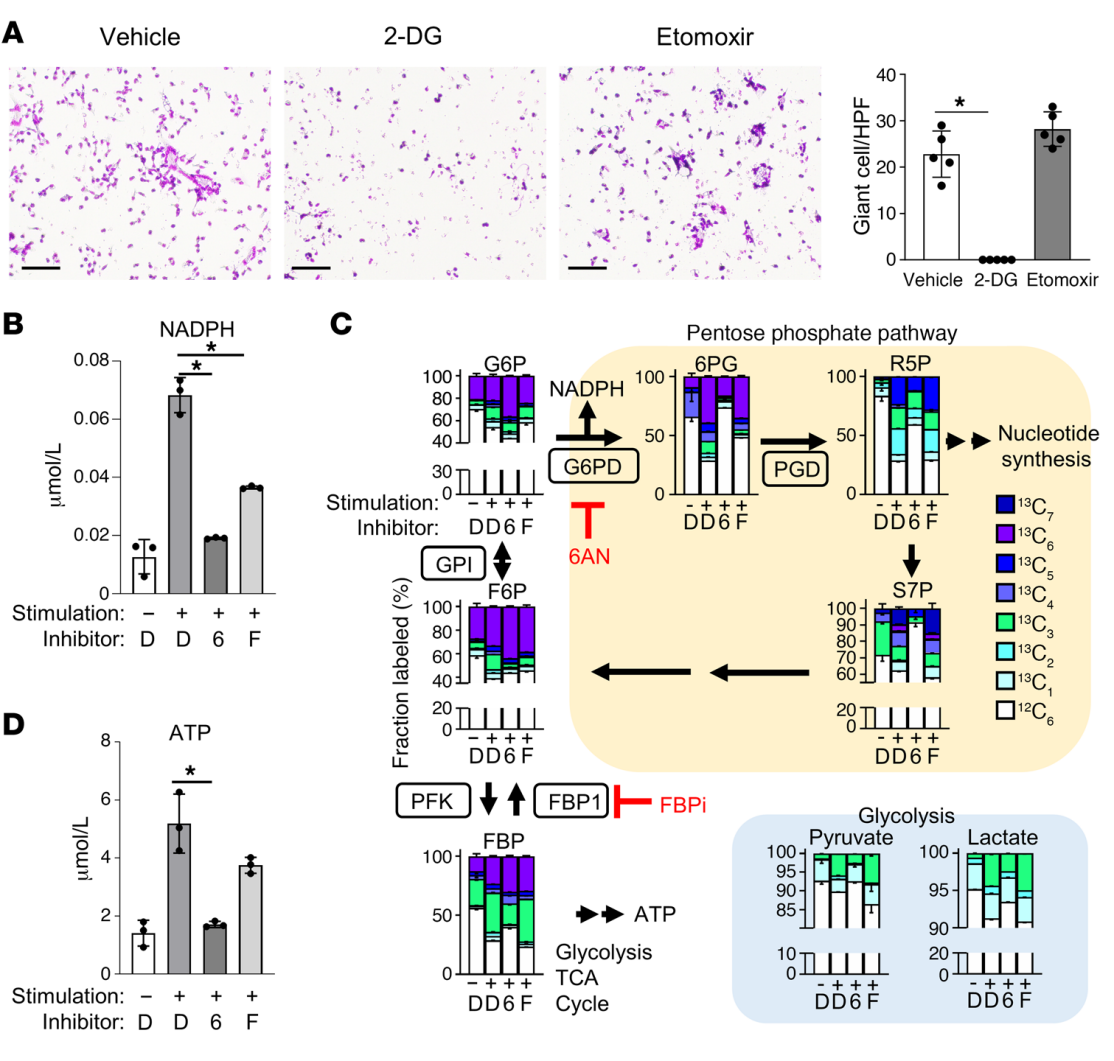
Metabolic analysis of in vitro giant cell model. (**A**) Giemsa staining (left) and bar graphs (right) for monocytes stimulated with Con A, IFN-γ, and anti-CD40 antibodies, with or without vehicle, 2-DG, and etomoxir (*n* = 5). Scale bars: 100 μm. Data are represented as mean ± SD. *P < 0.05, Dunnett’s multiple-comparisons test. Data are representative of at least 3 independent experiments. (**B**–**D**) Bar graph of analysis by MS of NADPH (**B**) and metabolites associated with PPP (**C**) and ATP (**D**) 1 hour after addition of ^13^C_6_ glucose to a giant cell model cultured for 3 days (*n* = 3). Data are represented as mean ± SD. *P < 0.05, unpaired *t* test. D, DMSO; 6, 6AN; F, FBPi.

The findings from scRNA-Seq showed that, apart from PPP, other metabolism-related genes were also upregulated in TREM2 macrophages in sarcoidosis (Figure 4E). To explore the roles of the glycolytic system and β-oxidation in giant cell formation, we added 2-deoxy-d-glucose (2-DG), a glycolytic system inhibitor, and etomoxir, a β-oxidation inhibitor, to an in vitro granuloma model. The data showed that 2-DG effectively halted giant cell formation, while etomoxir had no significant influence on this process, suggesting that giant cell formation may not rely on β-oxidation (Figure 7A). Conversely, given that PPP is a subsidiary pathway of the glycolytic system, the inhibition of this system might affect PPP. This assumption is supported by the observation that 2-DG inhibits downstream FBP1 (29).

Given that NADPH is generated not only via PPP but also through the mitochondrial electron transport chain (30), the effect of PPP inhibitors on NADPH production was investigated to ascertain whether NADPH synthesis in giant cells predominantly relies on the PPP. The amount of NADPH increased upon stimulation with IFN-γ, anti-CD40 antibody, and Con A, but decreased with the addition of 6AN and FBPi in this in vitro granuloma model (Figure 7B).

To perform a comprehensive analysis of glucose metabolites, peripheral blood monocytes were cultured with IFN-γ, anti-CD40 antibody, and Con A in combination with inhibitors of the PPP for 3 days. Subsequently, ^13^C_6_ glucose was added to the macrophages, and their metabolites were analyzed by MS 1 hour later. The use of glucose-containing ^13^C allows the measurement of glucose metabolites that are newly incorporated into cells. Compared with the control, an increase in ^13^C_6_ glucose–derived ^13^C influx following stimulation was detected (Figure 7C). The addition of 6AN resulted in a decrease in the fraction of ^13^C-labeled isotopomer of the PPP metabolites, specifically after 6-phosphogluconic acid (6PG). The addition of FBPi also reduced the ^13^C-labeled fraction of 6PG, indicating that the flux of NADPH-producing reactions is decreased. Conversely, FBPi exhibited a minimal effect on the PPP metabolites involved in nucleic acid de novo synthesis (Supplemental Figure 6). 6AN inhibited glycolytic systems such as pyruvate, lactate, and ATP production, whereas FBPi showed a mild inhibitory effect on the glycolytic system (Figure 7, C and D). These results indicate that even partial inhibition of the PPP by FBPi can inhibit giant cell formation by blocking NADPH production from macrophages. Depending on the degree of the PPP inhibition, giant cell formation may be inhibited without affecting nucleic acid and ATP synthesis.

### Inhibition of the PPP inhibits granuloma formation in an in vivo mouse model.

**Figure 8:**
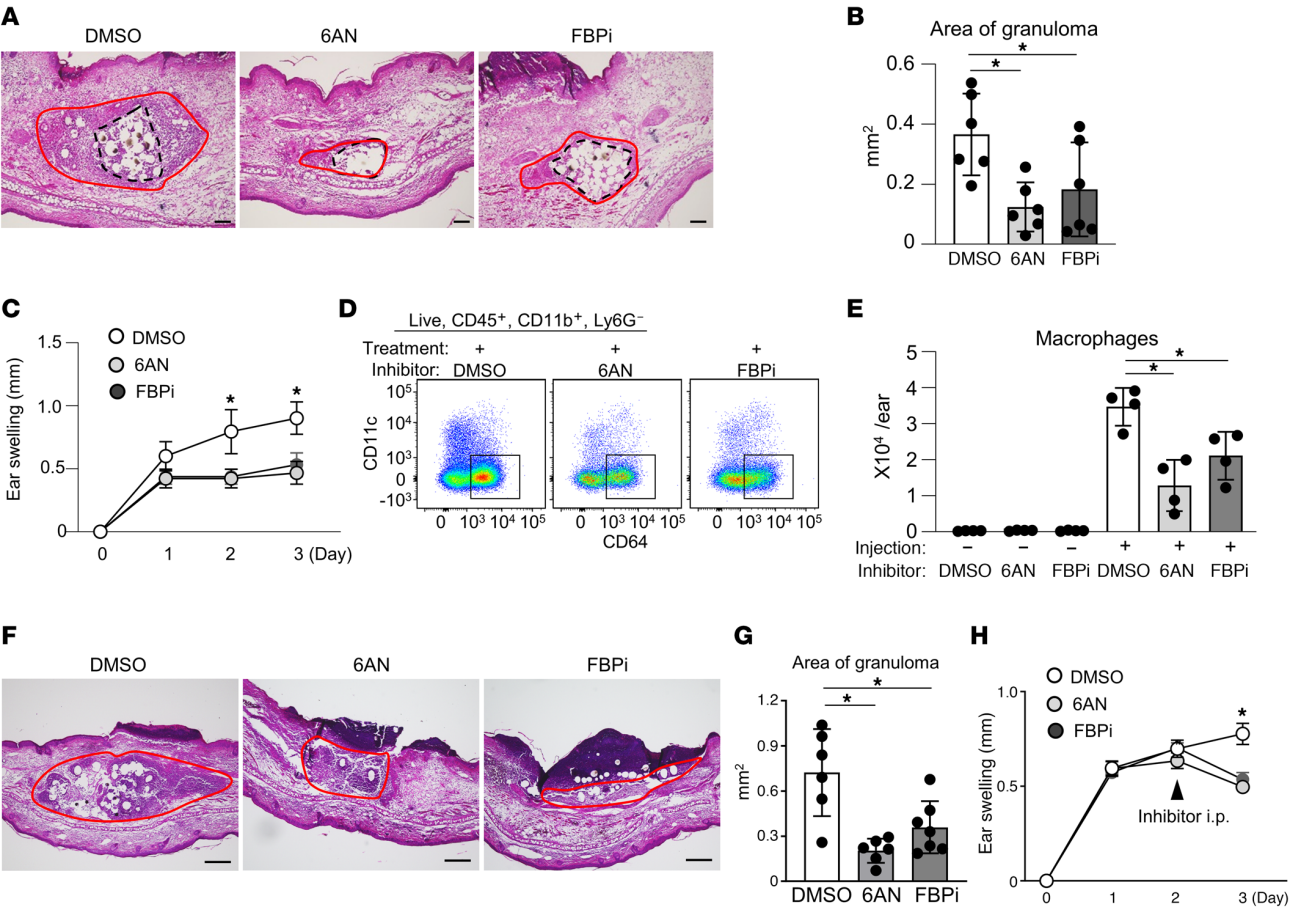
PPP inhibition reduces in vivo granuloma formation. (**A** and **B**) Representative photographs (**A**) and bar graph (**B**) of H&E-stained sections of ear skin from DMSO-, 6AN-, and FBPi-treated mice with Con A and bead injection (*n* = 6). Red lines indicate granulomas, and dotted lines indicate beads. Scale bars: 100 μm. (**C**) Time course of ear-swelling response (*n* = 6). (**D** and **E**) Dot plot and bar graph of flow cytometric analysis of the number of macrophages in the whole ear skin before and 72 hours after treatment (*n* = 4). (**F** and **G**) Representative photographs (**F**) and bar graphs (**G**) of H&E-stained sections of mouse ear skin (*n* = 6). Two days after subcutaneous injection of Con A and beads into mouse ear, DMSO, 6AN, or FBPi was administered intraperitoneally. Red lines indicate granulomas. Scale bars: 500 μm. (**H**) Time course of ear-swelling response (*n* = 6). Data are represented as mean ± SD. *P < 0.05, Dunnett’s multiple-comparisons test. Data are representative of at least 3 independent experiments.

We next determined whether the PPP is involved in granuloma formation in vivo using a murine granuloma-formation model by subcutaneous injection of polyacrylamide microbeads and Con A (31–33). WT mice were intradermally injected with Con A and polyacrylamide microbeads in the ear, which induced granuloma formation around the beads 3 days later (Supplemental Figure 7). Pretreatment with 6AN or FBPi reduced the area of granulomas in vivo (Figure 8, A and B). The thickness of the ear, an indicator of skin inflammation, was also attenuated by 6AN and FBPi administration (Figure 8C). Flow cytometry analysis of skin tissue showed that the number of macrophages infiltrating the skin was reduced by administration of 6AN and FBPi (Figure 8, D and E).

Furthermore, to better emulate a clinical scenario, we conducted an in vivo experiment in which mice were administered 6AN and FBPi 3 days after injection of Con A and microbeads. This experiment resulted in a noticeable reduction in granuloma size in the skin of inhibitor-treated mice (Figure 8, F and G). Histological examination disclosed necrosis of granuloma cells surrounding the beads in the group treated with PPP inhibitors, suggesting a potential increase in cell death. This outcome is congruent with findings from in vitro models using PPP inhibitors (Supplemental Figure 5B). Additionally, a reduction in ear swelling was observed a day after PPP inhibitor administration (Figure 8H).

These results indicate that inhibition of the PPP suppressed granuloma formation in the in vivo mouse model. Collectively, these findings suggest that inhibition of the PPP attenuates granulomas in both in vitro giant cell and in vivo granuloma models, indicating that the PPP may be a promising therapeutic target for granulomatous diseases.

## Discussion

Granulomatous disorders are ailments for which an established treatment regimen has yet to be identified due to the uncertain etiology. Here, we demonstrated that FBP1-positive TREM2 macrophages are increased in human sarcoidosis lesions and that serum FBP1 levels are increased in sarcoidosis patients. A subset of FBP1-positive TREM2 macrophages (TREM2_2 macrophages) showed enhanced activity of the PPP and gene expression similar to that of giant cells. In addition, the suppression of the PPP leads to the diminution of NADPH synthesis and the attenuation of giant cell and granuloma formation in both an in vitro giant cell model and an in vivo murine granuloma model. To the best of our knowledge, the current investigation represents the first examination of macrophage subsets within granulomas and the therapeutic intervention of granulomas via metabolic inhibition. Although the direct involvement of the PPP in human in vivo sarcoidosis formation has yet to be established, the results of our study demonstrate the improvement of granuloma formation through PPP inhibition. This suggests that the regulation of macrophage metabolism holds potential implications for the management of granulomatous diseases.

Our scRNA-Seq results pinpointed TREM2 macrophages in granulomas with pronounced FBP1 expression. Conversely, immunostaining illustrated diminished FBP1 expression in granuloma-residing giant cells relative to TREM2 macrophages. Moreover, both immunostaining and MS underscored the heightened expression of PPP-related enzymes in TREM2 macrophages and giant cells alike. This underscores the activation of the PPP pathway in both cell types, while uniquely amplifying FBP1 in TREM2 macrophages. The enzymatic function of FBP1 facilitates the conversion of FBP to F6P. While F6P transforms to G6P, it is worth noting that G6P is not solely directed to PPP but can also be converted to glucose or to glycogen. Previous studies have shown that neutrophils undergo glycogenesis during inflammation (34). By storing glycogen, energy can be produced even under conditions of reduced glucose and oxygen, such as at infection sites (35). Similarly in granulomas, peripherally situated TREM2 macrophages not only champion the PPP pathway but also bolster intracellular glucose and glycogen reserves via FBP1 upregulation. In contrast, centrally anchored giant cells may tap into these reserves to sustain the PPP pathway, offsetting potential nutrient deficiencies in the vicinity.

Recently, another research effort focusing on scRNA-Seq in cutaneous sarcoidosis was reported (36). The study underscored the existence of distinct macrophage subtypes in cutaneous sarcoidosis, labeled as homeostatic and granuloma-associated macrophages (GA). The GA population was further bifurcated into GA0 and GA1, with GA0 expressing ACE, CHI3L1, and other markers that closely match our identified TREM2 macrophages. Conversely, the GA1 macrophages exhibited an augmented expression of CXCL2 and VEGFA, resembling our resident macrophages. The authors additionally reported an increased presence of HIF1A and hypoxia-related genes in granuloma-associated macrophages. Notably, we too discerned the activation of HIF1A in our TREM2_2 macrophages, indicating a shared transcriptional response of macrophages in sarcoidosis patients. These congruent findings collectively suggest that the macrophage profiles we identified may have a wider relevance and universal significance for understanding of sarcoidosis pathogenesis. Importantly, our study’s focus on PPP activation in sarcoidosis macrophages introduces what we believe to be a previously unexplored facet of sarcoidosis pathobiology. As far as we are aware, this specific finding has not been previously reported and constitutes a unique contribution from our study.

There was an observed increase in TREM2 macrophages within sarcoidosis lesions. Previous studies have demonstrated that TREM2 expression is generally low in tissue-resident macrophages, including those in lymph nodes, while it is elevated in macrophages infiltrating tissues from the bloodstream (37). Moreover, granuloma induction was observed in fresh monocytes within granuloma models but not in macrophages differentiated in vitro or isolated from tissues (38, 39). This suggests that TREM2 macrophages are not prevalent in steady-state tissues and likely arise from monocytes recruited from the peripheral blood during specific inflammatory conditions. Our data affirm that TREM2 macrophages are infrequently found in healthy tissues.

The PPP is elevated in cancer cells and plays a crucial role in the proliferation and survival of cancer cells (40). Therefore, the PPP, especially G6PD, has attracted attention as a promising target for cancer therapy. Despite this, no inhibitors of the PPP have been implemented in a clinical setting to date due to the adverse effects of complete inhibition, including neurotoxicity and hemolysis as a result of oxidative stress (41, 42). The present study, through MS, demonstrates that suppression of FBP1 has a minimal effect on nucleic acid and ATP synthesis compared with suppression of G6PD, despite the suppression of NADPH production from the PPP. FBP1 inhibition does not induce hemolytic anemia or neurotoxicity (43). Therefore, FBP1 inhibition may serve as a therapeutic agent for sarcoidosis with fewer side effects.

In this study, we report on the presence of TREM2_2 macrophages, a subset of macrophages that are crucial for giant cell formation. These macrophages express genes such as cathepsin B and DC-STAMP, commonly found in osteoclasts, prototypical giant cells. However, it has yet to be discovered how the expression of these genes is regulated. The most studied giant cells are osteoclasts and foreign body giant cells. DC-STAMP, which is important for cell fusion, is known to be induced by the binding of c-Fos and nuclear factor of activated T cells, cytoplasmic 1 (NFATc1) in osteoclasts and PU.1 and NF-κB in foreign body giant cells to the promoter region of DC-STAMP (44). The TREM2_2 macrophages found in this study showed elevated expression of PU.1 and NF-κB, suggesting that giant cell formation occurs through a mechanism similar to that in foreign body giant cells. On the other hand, STAT1 expressed in TREM2_2 macrophages inhibits the formation of both osteoclasts and foreign body giant cells by suppressing STAT6 (45). The discrepancy in STAT1 expression may account for the difference between Langhans-type giant cells formed in sarcoidosis and foreign-body giant cells. For instance, cathepsin B, which is expressed in osteoclasts, is expressed in Langhans-type giant cells but not in foreign body giant cells (44).

Our findings indicate that TREM2_2 macrophages exhibit PPP activation and that the inhibition of PPP serves to inhibit the granuloma formation in an in vitro giant cell and in vivo granuloma model. However, the detailed mechanism has remained to be fully elucidated. In vitro, the inhibition of NOX, which produces ROS from NADPH, inhibited granuloma formation, suggesting that ROS plays a crucial role in granuloma formation. For example, ROS is shown to stimulate receptor activators of RANKL expression in osteoblasts to promote osteoclastogenesis (46). However, in the present scRNA-Seq analysis of sarcoidosis, RANKL-expressing cells were not detected (data not shown), and much remains to be understood about granulomatous responses that are not mediated by RANKL.

The PPP has been identified as a target for the treatment of sarcoidosis. Currently, the only approved treatment for sarcoidosis is glucocorticoid-based therapy (3). However, this treatment is not optimal, as it requires long-term administration and is associated with side effects such as hyperglycemia, immunosuppression, and osteoporosis. TNF-α inhibitors have also been studied, but their efficacy varies from patient to patient, with some studies showing no benefit. Janus kinase inhibitors currently in clinical trials have been reported to improve symptoms in patients with sarcoidosis (5). However, these inhibitors are also immunosuppressive and may increase the risk of infection and carcinogenesis. Inhibition of PPPs, particularly FBP1, may be a new therapeutic target and a potential means of reducing side effects.

In conclusion, we have demonstrated that the expression of genes associated with the PPP pathways is upregulated in granulomatous diseases such as sarcoidosis and that inhibiting PPP activity suppresses the granulomatous response. The PPP may be a promising target for developing therapeutics for sarcoidosis and other granulomatous diseases recalcitrant to current options.

## Methods

### Human blood and skin samples.

Skin biopsies (4 mm) were taken from 5 healthy subjects and 3 patients with sarcoidosis (Supplemental Table 1), and blood was collected from 32 healthy subjects and 14 patients with sarcoidosis. Human skin sections were obtained from healthy donors and patients with atopic dermatitis, psoriasis, sarcoidosis, granuloma annulare, lupus miliaris disseminatus faciei, or necrobiosis lipoidica at Kyoto University Hospital.

### Processing of human skin samples.

For each skin sample, a 4 mm punch biopsy was obtained after local anesthesia and was placed immediately into 1 mL BAMBANKER freezing medium (Nippon Genetic Co.) on ice and stored at –80°C until analysis (storage time was from 4 to 5 months). For cell dissociation, cryopreserved tissue was thawed in a 37°C water bath, transferred to 0.25% Trypsin-EDTA (Gibco, Life Technologies), and incubated at 37°C for 15 minutes. The tissue was then transferred to a dissociation solution for a second round of dissociation using the abovementioned procedure, followed by dissociation with a Miltenyi Human Whole Skin Dissociation Kit (Miltenyi Biotec) for 2.5 hours according to the manufacturer’s protocol. The tissue was manually disaggregated by pipetting up and down 10 times in a 1 mL wide bore pipette. Next, cells were passed through a 70 μm cell strainer (352340, Corning) and stored on ice. Cells were pelleted at 300*g* for 10 minutes and resuspended in 1 mL RPMI. Live cells were counted using 0.4% Trypan Blue (Gibco, Thermo Fisher Scientific; 15250061). Isolated cells were loaded onto a Chromium instrument (10 Genomics) according to the Chromium Single Cell 3′ Reagent Kit, version 3, user guide (https://support.10xgenomics.com/single-cell-gene-expression/library-prep/doc/user-guide-chromium-single-cell-3-reagent-kits-user-guide-v3-chemistry).

### scRNA-Seq sequencing and alignment.

Sample libraries were sequenced at a depth of over 20,000 reads per cell using DNBSEQ-G400 (MGI). The CellRanger pipeline (version 3.1.0) (10x genomics) was used to generate count matrices from FASTQ files using default parameters.

R (version 4.1.0) and the Seurat R package (version 4.0.6) (47) were used to analyze the scRNA-Seq data further. Cells with less than 200 or more than 4,000 genes detected or more than 20% mitochondrial gene expression were filtered out. Doublet cells were further removed using scDblFinder (version 3.16) (48). For each sample, counts were transformed and normalized using SCTransform with default thresholds, and integration of the samples was performed on the filtered and normalized objects. Principal component analysis (PCA) was performed on the integrated object using RunPCA. The number of significant principal components (PCs) (ndims [number of dimensions] = 30) was determined based on the ElbowPlot and DimHeatmap generated in Seurat. Nonlinear dimensional reduction and visualization were performed using RunUMAP (uniform manifold approximation and projection). The FindAllMarkers function on the normalized RNA assay, as recommended by Seurat, was used to find cluster markers that were used to annotate cell subsets manually. This process was used for the subclustering of individual cell subsets. Clusters defined exclusively by mitochondrial genes were of low quality and were subsequently removed before cluster marker analysis.

The myeloid clusters from the scRNA-Seq data from a variety of inflammatory skin diseases, including atopic dermatitis (16), psoriasis (16), granuloma annulare (17), leprosy (18), and sarcoidosis, and healthy skin were analyzed. The frequency of cells positive for *ACE* and *FBP1* was calculated, and the percentages of *ACE* and *FBP1*^+^ cells in each disease were calculated relative to the total number of myeloid cells in that disease.

### Pathway enrichment analysis.

A list of output genes by Seurat differential expression analysis with log_2_FC > 0.25 and adjusted to *P* < 0.05 was used to identify enrichment in macrophage clusters. Reactome analysis was performed using ReactomePA (version 1.38.0) (49).

### Receiver operating characteristic curve.

The receiver operating characteristic curve was plotted using pROC (version 1.18.0) (50).

### Immunofluorescence staining of human specimens.

Formalin-fixed, paraffin-embedded murine tissue was cut into 3 μm thick sections. Slides were stained automatically using a Leica Bond system (Leica Biosystems). Slides were deparaffinized, rehydrated, and washed with demineralized water before heat-induced antigen retrieval in BOND Epitope Retrieval 2 (Leica Biosystems) for 40 minutes. Protein blocking was achieved with Akoya Antibody Diluent/Block (Akoya Biosciences) for 10 minutes, followed by incubation with the first primary antibody for 1 hour, followed by the secondary antibody (Polymer HRP, Ms+ Rb; Akoya Biosciences) for 30 minutes, and finally, an Opal fluorophore (Akoya Biosciences) dissolved 1:50 in 1× Plus Amplification Diluent (Akoya Biosciences) for 10 minutes at room temperature. To facilitate multiplex staining with 3 markers, samples were heated for 10 minutes to facilitate antigen stripping. After this staining cycle, this procedure was repeated for 2 different primary antibodies, the secondary antibody, and corresponding Opal fluorophores. Finally, DAPI was used as a nuclear counterstain, and slides were mounted with ProLong Diamond Antifade Mountant (Life Technologies). All incubation steps were performed at room temperature. The primary antibodies were anti-CD68 (PG-M1, 1/100, Agilent Technologies-DAKO), anti-CD163 (10D6, 1/200, Leica), anti-FBP1 (EPR4619, 1/100, Abcam), anti-TREM2 (D814C, 1/100, Cell Signaling Technology), anti-CD1C (EPR23189-196, 1/1000, Abcam), anti-G6PD (EPR20668, 1/2000, Abcam), and anti-CHI3L1 (EPR19078-157, 1/250, Abcam). Images were captured using a BIOREVO BZ-9000 system (Keyence) and analyzed with ImageJ software (NIH).

### Image analysis.

QuPath software (version 0.3.2) was used for identifying cells and measuring the immunofluorescence intensity of each channel (51). First, the “Cell detection” function identified the cells based on the intensity of DAPI staining. Detected cells as detection objects were converted to annotation objects. Subsequently, these annotations were applied to other immunofluorescence images. To measure the mean intensity of each cell in the immunofluorescent staining images, we utilized the “Add intensity features” function in QuPath. We manually defined the threshold for positive cells on each staining. Following the measurement of fluorescent intensities in all detected cells and the determination of positive cells, the percentage of targeted populations was calculated for each image.

### ELISA.

Serum was collected from age-matched patients with sarcoidosis and healthy subjects and stored at –20°C. Serum FBP1 levels were measured using a Human FBP1 ELISA Kit (LS Bio). In addition, serum ACE was measured using previously reported methods (LSI Medience) (52).

### In vitro granuloma model.

Blood was collected from healthy volunteer donors. Peripheral blood mononuclear cells were isolated from whole blood using Lymphocyte Separation Solution (Nacalai Tesque) and incubated with anti-CD14 microbeads (Miltenyi Biotec). CD14^+^ cells were isolated using an AutoMACS Benchtop Magnetic Cell Sorter according to the manufacturer’s protocol (Miltenyi Biotec). The purity of isolated cells was greater than 95%, as demonstrated by flow cytometry using a FACS Fortessa System (BD Biosciences). Isolated CD14^+^ monocytes were resuspended at a density of 5 × 10^4^ cells per well in 96-well plates in RPMI-1640 medium (Gibco, Thermo Fisher Scientific) containing 10% fetal bovine serum, 2 mM l-glutamine, 50 μM β-mercaptoethanol, 100 U/mL penicillin, and 100 mg/mL streptomycin (cRPMI). Cells were then cultured for 72 hours with 5 mg/mL Con A (Nacalai Tesque), 10 ng/mL IFN-γ (PeproTech), and 1 mg/mL anti-CD40 antibody (BioLegend). For giant cell treatment, giant cell formation was evaluated after 3 days of incubation with Con A, IFN-γ, and anti-CD40 antibody, followed by the addition of the inhibitor and incubation for another 3 days. At the end of the culture period, cells were treated with Giemsa stain to detect nuclei. Giant cells were defined as cells with more than 3 nuclei per cell, according to definitions established by previous studies (25). The stained plates were examined using an Olympus CKX53 microscope. For cell-viability assessment, the cells were harvested and stained with Trypan Blue (Thermo Fisher Scientific) to determine the percentage of nonviable (dead) cells. Intracellular NADPH was measured using the NADP/NADPH Assay Kit (Dojindo). Supernatants from cell cultures were analyzed using a Cytometric Bead Array (BD Biosciences), following the manufacturer’s recommended protocol. The bead fluorescence was assessed with the LSR Fortessa instrument (BD Biosciences), and the data were analyzed using FCAP Array, version 3.0, software (BD Biosciences). For PPP or metabolic inhibition, 6AN (10 μM, Sigma-Aldrich), FBPi (100 μM, Cayman Chemical), G6PDi (50 μM, Cayman Chemical), DPI (10 μM, Cayman Chemical), dimethyl sulfoxide, water, 2-DG (5 mM) (Wako), or etomoxir (250 μM) (Cayman Chemical) was added to the medium at the start of culture.

### Quantitative PCR analysis.

RNA was extracted directly from culture plates using an RNeasy Mini Kit (QIAGEN). cDNA was reverse transcribed from total RNA samples using a Prime Script RT Reagent Kit (Takara Bio). Quantitative reverse-transcriptase PCR (RT-qPCR) was performed by monitoring the synthesis of double-stranded DNA during the various PCR cycles with SYBR Green I (Roche) and a LightCycler real-time PCR apparatus (Roche) according to the manufacturer’s instructions. All primers were obtained from Greiner Japan. The primer sequences were as follows: ACTB, 5′-CCAACCGCGAGAAGATGA-3′ (forward) and 5′-CCAGAGGCGTACAGGGATAG-3′ (reverse); FBP1 5′-ACATCGATTGCCTTGTGTCC-3′ (forward) and 5′-CATGAAGCAGTTGACCCCAC-3′ (reverse); and G6PD 5′-ACGACGAAGCGCAGACAG-3′ (forward) and 5′-CCGACTGATGGAAGGCATCG-3′ (reverse). The cycling conditions were as follows: initial enzyme activation at 95°C for 10 minutes, followed by 45 cycles at 95°C for 10 seconds and 60°C for 20 seconds. Gene-specific fluorescence was measured at 60°C. For each sample, duplicate test reactions were analyzed for gene expression, and the results were normalized to those for the housekeeping gene ACTB.

### IC-MS–based metabolomic analysis of intracellular bacterial metabolites.

Peripheral blood monocytes were incubated with Con A, IFN-γ, and anti-CD40 antibody with PPP inhibitors for 3 days; then ^13^C_6_ glucose (Sigma-Aldrich) was added to the medium at a concentration of 2 mg/mL. One hour later, cells were washed twice with cold PBS, frozen by liquid nitrogen, and stored at –80°C until metabolite extraction. Frozen cells were lysed with methanol (500 μL) and added to half the volume of water and a 0.4 volume of chloroform (LC/MS grade, Wako). The suspension was centrifuged at 15,000*g* for 15 minutes at 4°C. After centrifugation, the aqueous phase was subjected to ultrafiltration using an ultrafiltration tube (Ultrafree MC-PLHCC, Human Metabolome Technologies). The filtrate was concentrated using a vacuum concentrator (SpeedVac, Thermo Fisher Scientific). The concentrated filtrate was dissolved in 50 μL ultrapure water and used for ion chromatography with MS (IC-MS) analysis. Methionine sulfone and 2-morpholinoethanesulfonic acid were used as internal standards for cationic and anionic metabolites, respectively. The loss of endogenous metabolites during sample preparation was corrected by calculating the recovery (%) of the standards in each sample measurement. Metabolites were measured using an orbitrap-type MS (Q-Exactive Focus; Thermo Fisher Scientific) connected to a high-performance IC system (ICS-5000+, Thermo Fisher Scientific), which allows highly selective and sensitive metabolite quantification based on the principle of IC separation and Fourier transfer MS (53). The IC was equipped with an anion-electrolytic suppressor (Thermo Scientific Dionex AERS 500) to convert the potassium hydroxide gradient to pure water before the sample entered the mass spectrometer. Separation was performed using a Thermo Scientific Dionex IonPac AS11-HC 4 mm particle size column. The IC flow rate was set at 0.25 mL/min, supplemented by a postcolumn make-up flow of MeOH at 0.18 mL/min. The potassium hydroxide gradient conditions for IC separation were set as follows: from 1 mM to 100 mM (0–40 minutes), 100 mM (40–50 minutes), and 1 mM (50.1–60 minutes) at a column temperature of 30°C. The Q Exactive Focus mass spectrometer was operated in ESI-negative mode for all analyses. A complete mass scan (*m/z* 70–900) was performed at a resolution of 70,000. The automatic gain control target was set to 3 × 10^6^ ions, and the maximum ion injection time was set to 100 msec. The source ionization parameters were optimized with a spray voltage of 3 kV, and other parameters were set as follows: transfer temperature = 320°C, S-lens level = 50, heater temperature = 300°C, sheath gas = 36, and aux gas = 10.

### Imaging MS.

Imaging MS using an ion trap instrument was performed as described (54). Briefly, frozen skin tissue sections (10 mm) mounted on ITO-coated glass slides for MALDI-MS imaging were spray-coated with a 9-aminoacridine matrix (10 mg/mL in 80% ethanol). Mass spectra were acquired using a matrix-assisted laser desorption/ionization (MALDI) linear ion trap mass spectrometer (MALDI LTQ XL; Thermo Fisher Scientific) equipped with a 60 Hz N2 laser. The laser scan pitch was 35 mm, and each pixel was irradiated 50 times at a repetition rate of 20 Hz. MS was run in negative ion detection mode, and the ion signal at *m/z* 744 corresponding to NADPH was recorded using selective ion monitoring. Data were imaged using ImageQuest software, version 1.0.1 (Thermo Fisher Scientific).

### Mouse models.

Female C57BL/6 mice were obtained from Oriental Bioservice Inc. DMSO, 6AN (500 mg/mouse), or FBPi (1.5 mg/mouse) was administered intraperitoneally to mice 6 hours before or 48 hours after intradermal injection of Con A (25 mg) and 2.5 × 10^4^ polyacrylamide microbeads (Bio-gelP-100; Bio-Rad) in PBS into the dorsal ear (55). Ear swelling was evaluated by measuring ear thickness with a caliper (Mitutoyo) before and 24, 48, and 96 hours after injection.

### Flow cytometry.

Skin from the ear of the mice was collected 0 and 72 hours after injection, and the dorsal halves were floated for 80 minutes at 37°C on 0.33 mg/mL of Liberase TL (Roche) and 0.5 mg/mL DNAase (Sigma-Aldrich) dissolved in cRPMI. The sample was filtered through a 70 μm cell strainer mesh (Thermo Fisher Scientific).

Isolated cells were stained with the following: antibodies against mouse CD45 (30-F11; BD Biosciences), CD4 (RM4-5; BD Biosciences), CD8b (H35-17.2; Invitrogen), CD11b (M1/70; BD Biosciences), CD11c (N418; BioLegend), CD64 (X54-5/7.1; BD Biosciences), CD90.2 (53-2.1; BioLegend), Ly6C (HK1.4; BioLegend), CD16/32 (2.4G2; BD Biosciences), Ly6G (1A8; BD Biosciences), and MHC class II (M5/114.15.2; Invitrogen); and dead cell identification dye (Fixable viability dye; Invitrogen). Flow cytometry was performed using LSR Fortessa (BD Biosciences) and analyzed with FlowJo software, version 10.7.1 (BD Biosciences).

### Histology and immunofluorescence staining of mouse specimens.

For histological examination, mouse skin samples were fixed with 10% formalin in phosphate-buffered saline and embedded in paraffin. Sections (5 μm thick) were prepared and stained with H&E. For immunofluorescence staining, samples were immersed in 1% paraformaldehyde (Nacalai Tesque) overnight at 4°C, embedded in OCT compound (Sakura), frozen, and then sectioned. After treatment with Image-iT FX Signal Enhancer (Life Technologies), sections were incubated with anti-mouse CD68 (FA-11, BioLegend) and anti-FBP1 (EPR4619, 1/100, Abcam) overnight at 4°C and then with Alexa Fluor 594 anti-rabbit IgG and Alexa Fluor 647 anti-rat IgG (Life Technologies) for 30 minutes. Slides were mounted using ProLong Antifade with DAPI (Life Technologies). Images were captured on a fluorescent microscope (BZ-900, Keyence).

### Statistics.

Statistical analyses used for each experiment are specified in the figure legends. The significance of the quantification results was tested by Šidák’s multiple-comparisons tests (Figure 1D and Figure 4B), Dunnett’s multiple comparisons tests (Figure 2C, Figure 3B, Figure 6, E, G, and I; Figure 7A; and Figure 8), Tukey’s multiple comparisons tests, Mann-Whitney *U* tests (Figure 3C and Figure 4G), and Student’s *t* tests (Figure 6, A and B; Figure 7, B and D) using Prism 9.0 (GraphPad Software).

### Study approval.

Human samples were obtained by a protocol approved by the Kyoto University Graduate School and Faculty of Medicine Ethics Committee (reference no. R0743). All subjects provided written consent that was approved by the Institutional Review Board of Kyoto University. All animal procedures used in this study followed national guidelines. The Animal Research Committee of Kyoto University granted ethical approval and permission (MedKyo 22266).

### Data availability.

Values for all data points in graphs and values behind the reported means are provided in the Supplemental Supporting Data Values file. scRNA-Seq data were deposited in the NCBI’s Gene Expression Omnibus database (GEO GSE234901).

## Author contributions

S Nakamizo and KK conceived the project. S Nakamizo, YS, YI, YU, AY, MK, and KK performed experiments. S Nakamizo, YI, YS, SY, MK, and KK performed formal analysis. S Nakamizo, YI, YS, and MK curated data. S Nakamizo, YS, YI, S Nakajima, NK, GE, and KK wrote the manuscript. S Nakamizo, YU, HT, HD, AY, TM, KM, MK, S Nomura, IK, SO, S Nakajima, NK, GE, and KK provided resources. S Nakamizo and KK acquired funding. KK supervised the project.

## Supplementary Material

Supplemental data

Supporting data values

## Version 1 12/01/2023

Electronic publication
