# Supplementary material for: Activation of the pentose phosphate pathway in macrophages is crucial for granuloma formation in sarcoidosis
Source: J Clin Invest. 2023 Dec 1;133(23):e171088. doi: 10.1172/JCI171088 (PMC10688990; doi:10.1172/JCI171088)
Supplement: Supplemental data [file jci-133-171088-s190.pdf]

Supplementary Methods

Analysis of nuclear transcription factors

A list of output genes by Seurat differential expression analysis with Log2FC>0.25 and adjusted to P<0.05 was used to identify enrichment in macrophage clusters. Metascape was used to estimate nuclear transcription factors(1).

Quantitative PCR analysis

RNA was extracted directly from mouse skin using an RNeasy mini kit (Qiagen, Hilden, Germany). cDNA was reverse-transcribed from total RNA samples using a Prime Script RT reagent kit (Takara Bio, Otsu, Japan). Quantitative RT-PCR was performed by monitoring the synthesis of double-stranded DNA during the various PCR cycles with SYBR Green I (Roche, Basel, Switzerland) and a LightCycler real-time PCR apparatus (Roche) according to the manufacturer's instructions. All primers were obtained from Greiner Japan (Tokyo, Japan). The primer sequences were: Actb 5'-CCC CAA CTT GAT GTA TGA AGG C-3' (forward) and 5'-TCA AGT CAG TGT ACA GGC CAG C-3' (reverse); Ifng 5'-CAG CAA CAG CAA GGC GAA AAA GG-3' (forward) and 5'- TTT CCG CTT CCT GAG GCT GGA T-3' (reverse); Lyz2 5'-TGAACGTTGTGAGTTTGCCA-3' (forward) and 5'-TGA GCT AAA CAC ACC CAG TCG-3' (reverse). The cycling conditions were: initial enzyme activation at 95 °C for 10 min, followed by 45 cycles at 95 °C for 10 sec and 60 °C for 20 sec. Gene-specific fluorescence was measured at 60 °C. For each sample, duplicate test reactions were analysed for gene expression, and the results were normalized to those for the housekeeping gene Actb.

Supplementary References

1. Zhou Y, Zhou B, Pache L, Chang M, Khodabakhshi AH, Tanaseichuk O, Benner C, and Chanda SK. Metascape provides a biologist-oriented resource for the analysis of systems-level datasets. *Nat Commun.* 2019;10(1):1523.

Supplementary Figure Legends

Supplementary Figure 1. Cell subsets observed in sarcoidosis lesions

(**A**) Violin plots of gene counts, RNA counts, and percentage of mitochondrial genes for each sample. (**B**) UMAP plot for total cells colored by cell subsets. FB: fibroblasts, KC: keratinocytes, TC: T cells, VEC: vascular endothelial cells, PC-vSMC: pericytes and vascular smooth muscle cells, APC: antigen-presenting cells, LEC: lymphatic endothelial cells, MEL: melanocytes, NC: nerve cells, and Prolif: proliferating cells. (**C**) UMAP plot separated by samples. (**D**) UMAP plots showing representative genes for each subset. The color scale represents the normalized gene expression level. (**E**) Heatmap showing three representative marker genes for each cell subset. (**F**) UMAP plots showing FN1, APOC1, APOE, and FBP1 genes for each subset. The color scale represents the normalized gene expression level.

Supplementary Figure 2. Sarcoidosis granulomas are composed of TREM2 macrophages

(**A**) Representative immunofluorescence staining (left) and bar graph (right) in healthy (n=3) and sarcoidosis (n=3) skin biopsy samples for expression of CD68 in gray, FBP1 in red, HLA-DR in green, and DAPI in blue. Scale bar = 100 μm. Šídák's multiple comparisons test was conducted between the healthy and sarcoidosis samples for each subset; $^*P < 0.05$. (**B**) Frequency of FBP1-positive and CD163-positive macrophages in skin biopsy samples from healthy subjects (n=3) and sarcoidosis (n=3). Šídák's multiple comparisons test was conducted between the healthy and sarcoidosis samples for each subset. (**C**)Representative immunofluorescence staining in sarcoidosis (n=3) skin biopsy

samples for expression of CD68 in gray, FBP1 in red, TREM2 in green, and DAPI in blue. Scale bar = 100 μm. (**D**) Frequency of FBP1^{+}/TREM2^{-}, FBP1^{+}/TREM2^{+}, FBP1^{-} /TREM^{2}- and FBP1^{-}/TREM2^{+} macrophages (CD68^{+}) in sarcoidosis skin. Tukey's multiple comparisons tests were conducted; *$P < 0.05$.

Supplementary Figure 3. Gene expression analysis of FBP1-positive macrophages

(**A**) Dot plot of genes upregulated in FBP1-positive macrophages in cardiac sarcoidosis in skin macrophage. (**B**) Bar graph of nuclear transcription factors expressed in TREM2_2 macrophages from gene expression in single-cell RNA-seq data. (**C**) Reactome terms among upregulated DEGs in TREM2_2 macrophages. The enriched terms were identified by adjusted P-value < 0.05.

Supplementary Figure 4. Activation of PPP in FBP1-positive macrophages

(**A**) Representative immunofluorescence staining in inflammatory skin disease samples (n=3) for expression of G6PD in gray, FBP1 in red, CD163 in green, and DAPI in blue. Scale bar = 100 μm. AD: atopic dermatitis, PSO: psoriasis. (**B**) Bar graph quantifying FBP1-positive, G6PD-positive cells in skin biopsy samples from healthy (n=3), inflammatory skin disease samples (n=3), and sarcoidosis samples (lung: n=3, heart: n=1, lymph node: n=3). P-values were obtained by Dunnett's multiple comparisons test; *$P < 0.05$. (**C**) Dot plot of NOX genes for each macrophage subset in all skin scRNA-seq data (5 healthy subjects and 3 patients). (**D**) Violin plots showing expression of NOX-related genes in each macrophage subset in all skin scRNA-seq data (5 healthy subjects and 3 patients).

Supplementary Figure 5. Effects of PPP inhibitors on cytotoxicity and inflammatory cytokines

(**A**) Evaluation of dead cell percentages post 3-day incubation of monocytes with Con A, IFN-γ, anti-CD40 antibody, and either DMSO or 6AN (n=5). (**B**) Percentage of dead cells in 3-day cultures primed with Con A, IFN-γ, and anti-CD40 antibodies, and subsequently incubated with either DMSO or 6AN for an additional 3 days (n=3-4). (**C**) Cytokine concentrations in the supernatant after incubation of monocytes with Con A, IFN-γ, and anti-CD40 antibodies in the presence of either DMSO (D), FBPi (F), or 6AN (6) for 3days. Scale bars = 100 μm. Results are expressed as the mean ± SD. Data are representative of at least three independent experiments. P-values were obtained by Dunnett's multiple comparisons tests; $*P < 0.05$.

Supplementary Figure 6. Effects of PPP inhibitors on nucleic acid synthesis

Bar graph of analysis by mass spectrometry of nucleic acid 1 hour after addition of $^{13}C_6$ glucose to a giant cell model cultured for three days. Results are expressed as the mean ± SD. P-values were obtained by Tukey's multiple comparisons tests; $*P < 0.05$. D: DMSO, 6: 6AN, F: FBPi.

Supplementary Figure 7. Mouse granuloma model with subcutaneous injection of Con A and beads

(**A**) Images of immunostained tissues three days after subcutaneous injection of Con A and beads to mouse skin (n=3). Green: FBP1, Red: CD68, Blue: DAPI. Scale bar = 50 μm. (**B**) Bar graph of quantitative PCR on mouse skin three days after subcutaneous

injection of Con A and beads (n=5). Data are representative of three independent experiments.

Supplementary Table 1. Sample summary

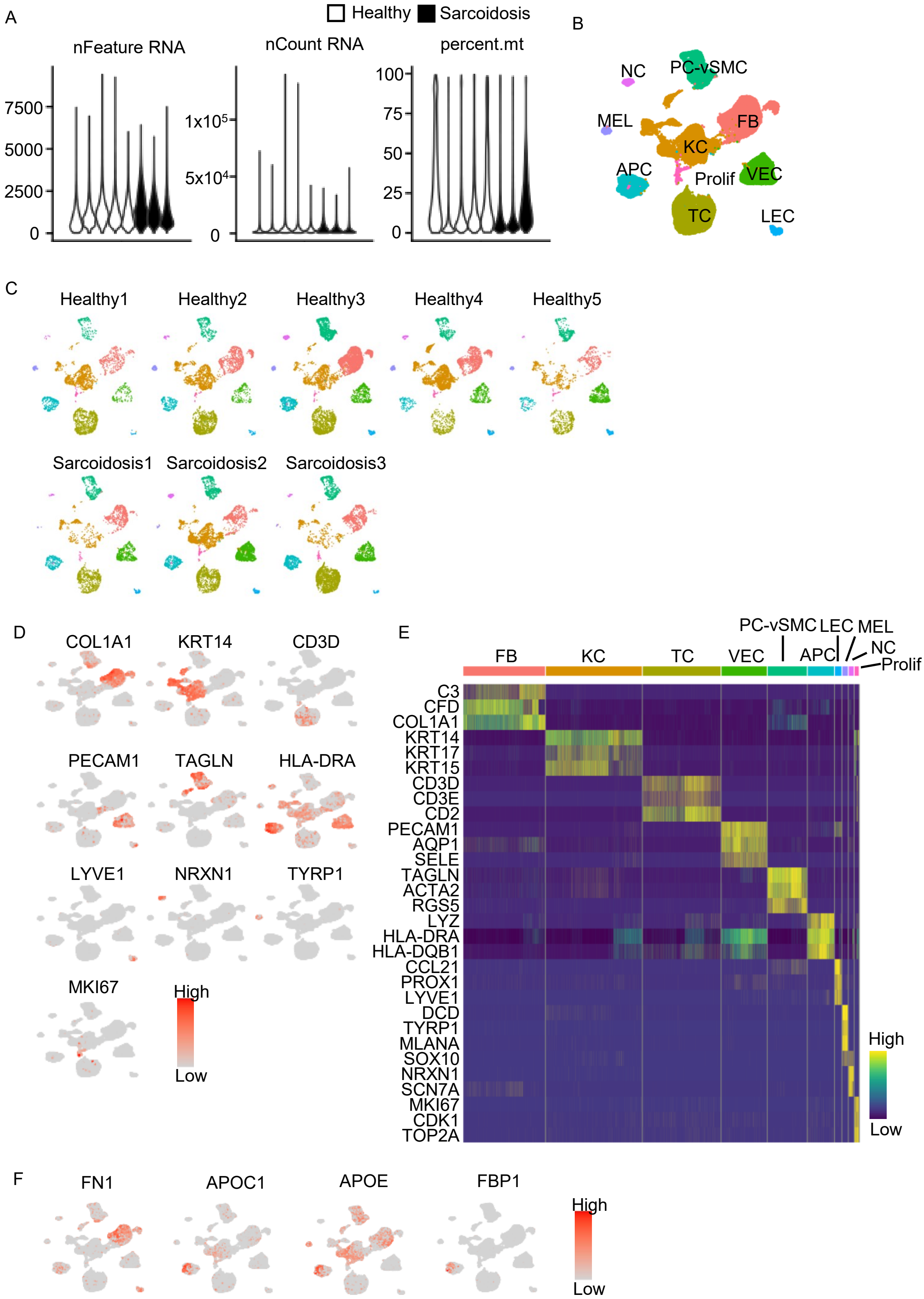

Supplementary Figure 1. Cell subsets observed in sarcoidosis lesions

(**A**) Violin plots of gene counts, RNA counts, and percentage of mitochondrial genes for each sample. (**B**) UMAP plot for total cells colored by cell subsets. FB: fibroblasts, KC: keratinocytes, TC: T cells, VEC: vascular endothelial cells, PC-vSMC: pericytes and vascular smooth muscle cells, APC: antigen-presenting cells, LEC: lymphatic endothelial cells, MEL: melanocytes, NC: nerve cells, and Prolif: proliferating cells. (**C**) UMAP plot separated by samples. (**D**) UMAP plots showing representative genes for each subset. The color scale represents the normalized gene expression level. (**E**) Heatmap showing three representative marker genes for each cell subset. (**F**) UMAP plots showing FN1, APOC1, APOE, and FBP1 genes for each subset. The color scale represents the normalized gene expression level.

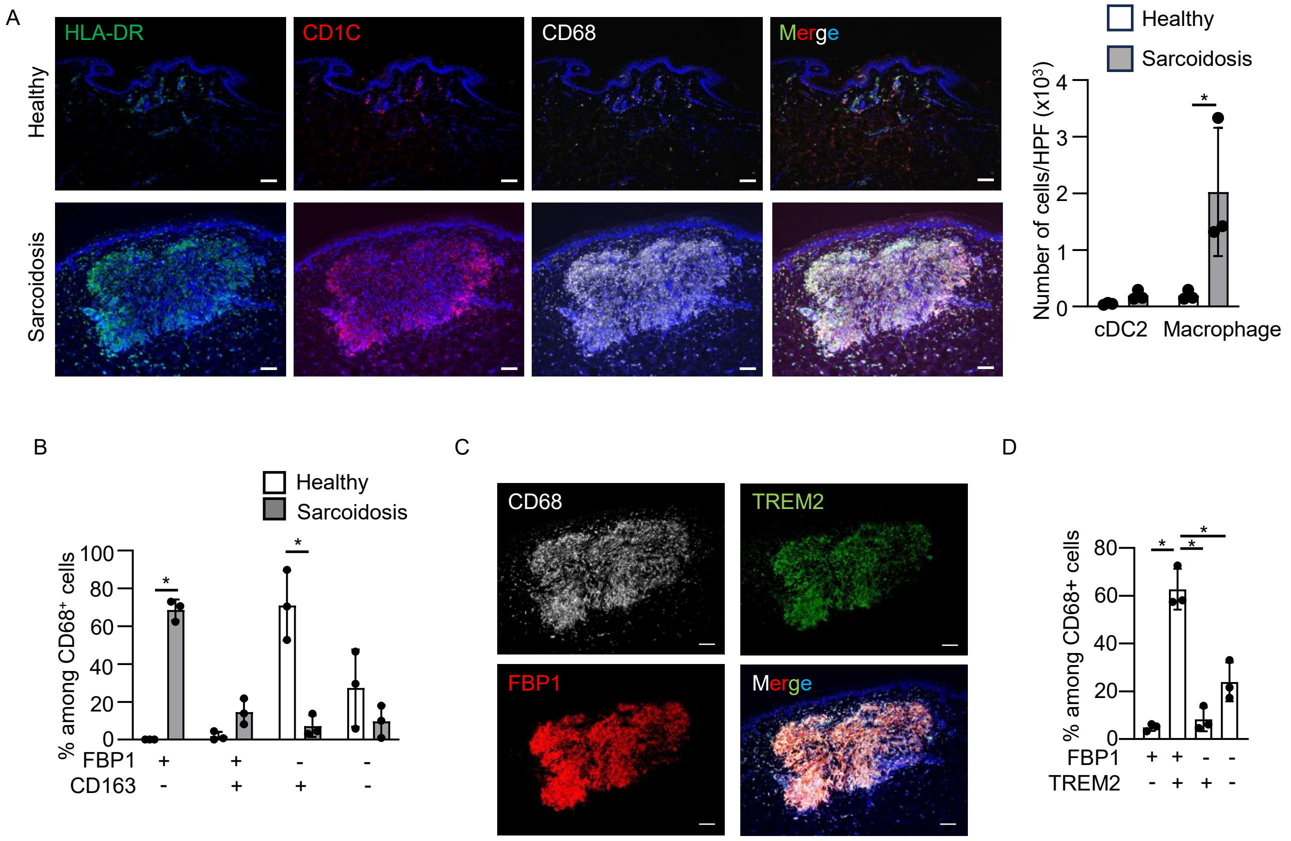

Supplementary Figure 2. Sarcoidosis granulomas are composed of TREM2 macrophages

(**A**) Representative immunofluorescence staining (left) and bar graph (right) in healthy (n=3) and sarcoidosis (n=3) skin biopsy samples for expression of CD68 in gray, FBP1 in red, HLA-DR in green, and DAPI in blue. Scale bar = 100 µm. Šídák's multiple comparisons test was conducted between the healthy and sarcoidosis samples for each subset; *$P < 0.05$. (**B**) Frequency of FBP1-positive and CD163-positive macrophages in skin biopsy samples from healthy subjects (n=3) and sarcoidosis (n=3). Šídák's multiple comparisons test was conducted between the healthy and sarcoidosis samples for each subset. (**C**)Representative immunofluorescence staining in sarcoidosis (n=3) skin biopsy samples for expression of CD68 in gray, FBP1 in red, TREM2 in green, and DAPI in blue. Scale bar = 100 µm. (**D**) Frequency of $FBP1^+/TREM2^-$, $FBP1^+/TREM2^+$, $FBP1^-/TREM2$- and $FBP1^-/TREM2^+$ macrophages ($CD68^+$) in sarcoidosis skin. Tukey's multiple comparisons tests were conducted; *$P < 0.05$.

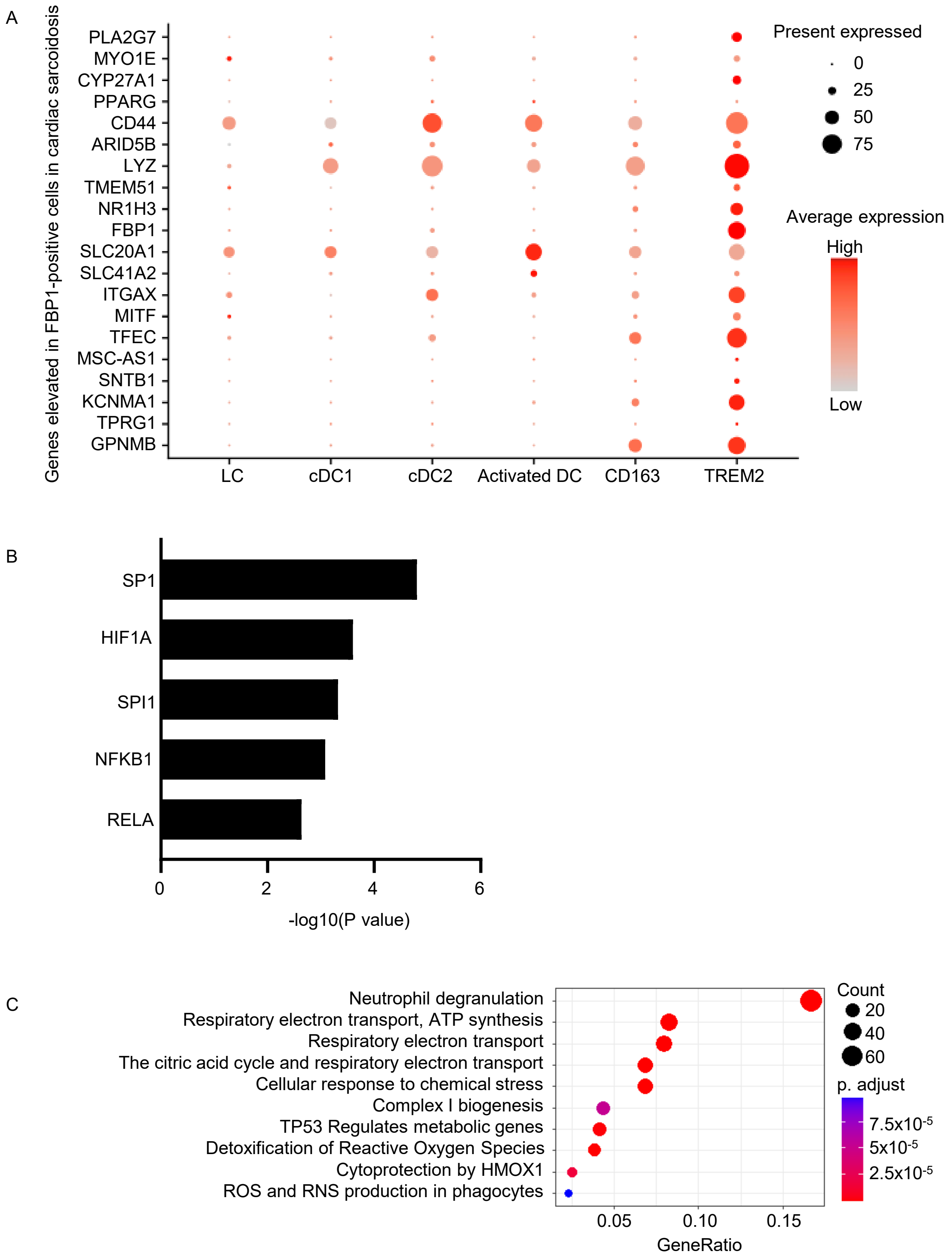

Supplementary Figure 3. Gene expression analysis of FBP1-positive macrophages

(**A**) Dot plot of genes upregulated in FBP1-positive macrophages in cardiac sarcoidosis in skin macrophage. (**B**) Bar graph of nuclear transcription factors expressed in TREM2_2 macrophages from gene expression in single-cell RNA-seq data. (**C**) Reactome terms among upregulated DEGs in TREM2_2 macrophages. The enriched terms were identified by adjusted P-value < 0.05.

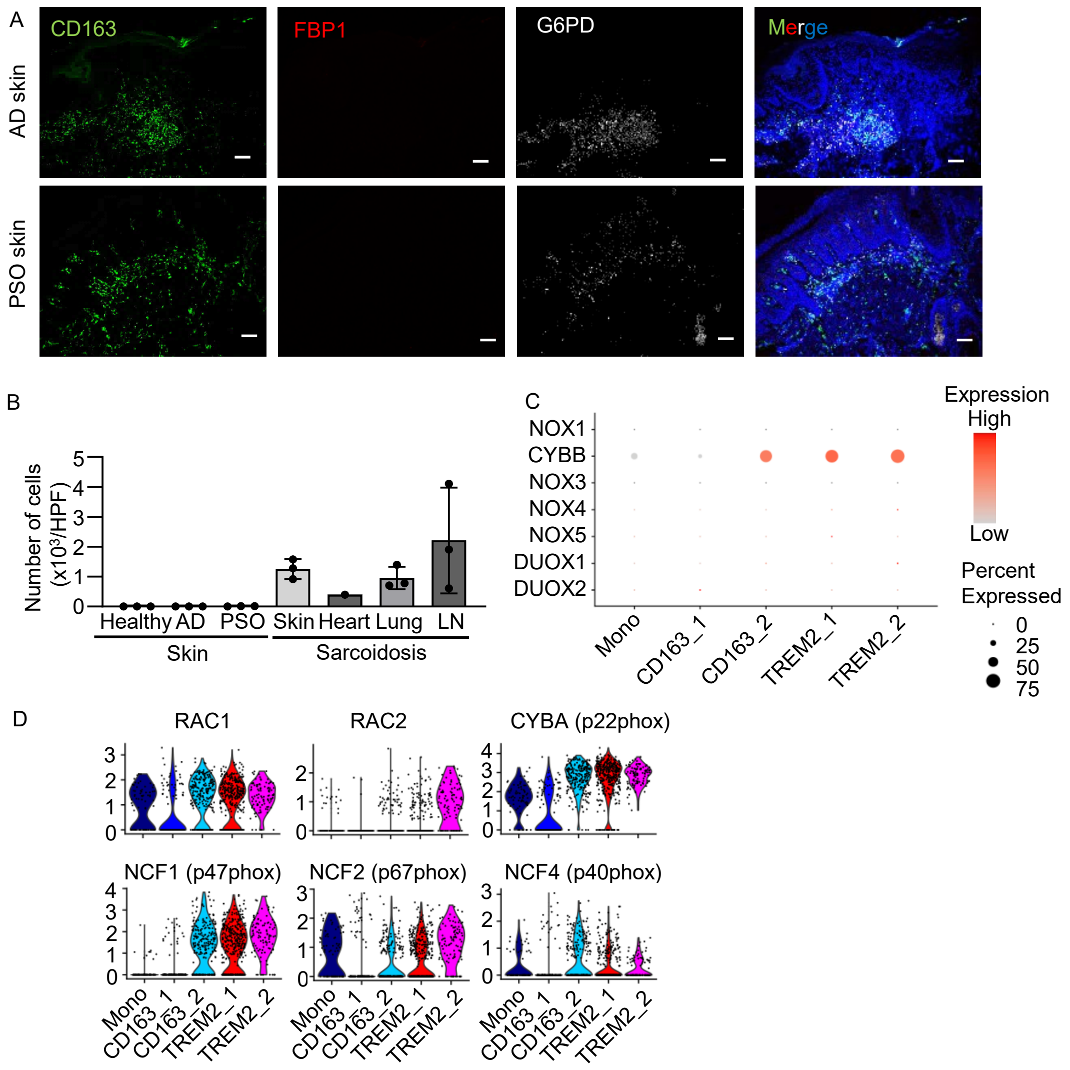

Supplementary Figure 4. Activation of PPP in FBP1-positive macrophages

(**A**) Representative immunofluorescence staining in inflammatory skin disease samples (n=3) for expression of G6PD in gray, FBP1 in red, CD163 in green, and DAPI in blue. Scale bar = 100 μm. AD: atopic dermatitis, PSO: psoriasis. (**B**) Bar graph quantifying FBP1-positive, G6PD-positive cells in skin biopsy samples from healthy (n=3), inflammatory skin disease samples (n=3), and sarcoidosis samples (lung: n=3, heart: n=1, lymph node: n=3). P-values were obtained by Dunnett's multiple comparisons test; *$P < 0.05$. (**C**) Dot plot of NOX genes for each macrophage subset in all skin scRNA-seq data (5 healthy subjects and 3 patients). (**D**) Violin plots showing expression of NOX-related genes in each macrophage subset in all skin scRNA-seq data (5 healthy subjects and 3 patients).

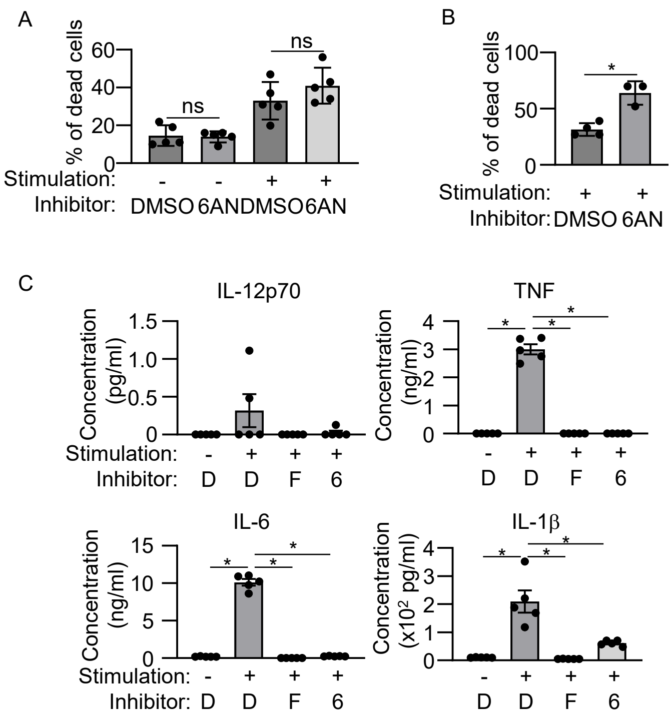

Supplementary Figure 5. Effects of PPP inhibitors on cytotoxicity and inflammatory cytokines

(**A**) Evaluation of dead cell percentages post 3-day incubation of monocytes with Con A, IFN-γ, anti-CD40 antibody, and either DMSO or 6AN (n=5). (**B**) Percentage of dead cells in 3-day cultures primed with Con A, IFN-γ, and anti-CD40 antibodies, and subsequently incubated with either DMSO or 6AN for an additional 3 days (n=3-4). (**C**) Cytokine concentrations in the supernatant after incubation of monocytes with Con A, IFN-γ, and anti-CD40 antibodies in the presence of either DMSO (D), FBPi (F), or 6AN (6) for 3days. Scale bars = 100 μm. Results are expressed as the mean ± SD. Data are representative of at least three independent experiments. P-values were obtained by Dunnett's multiple comparisons tests; *$P < 0.05$.

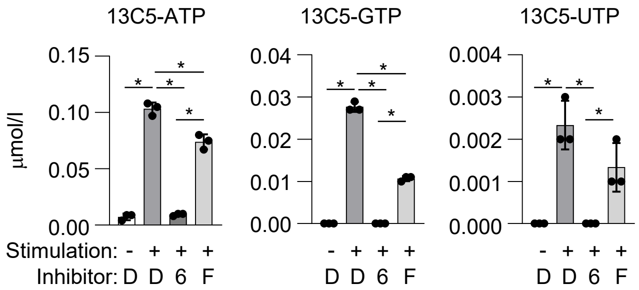

Supplementary Figure 6. Effects of PPP inhibitors on nucleic acid synthesis.

Bar graph of analysis by mass spectrometry of nucleic acid 1 hour after addition of $^{13}C_6$ glucose to a giant cell model cultured for three days. Results are expressed as the mean ± SD. P-values were obtained by Tukey's multiple comparisons tests; *P < 0.05. D: DMSO, 6: 6AN, F: FBPi.

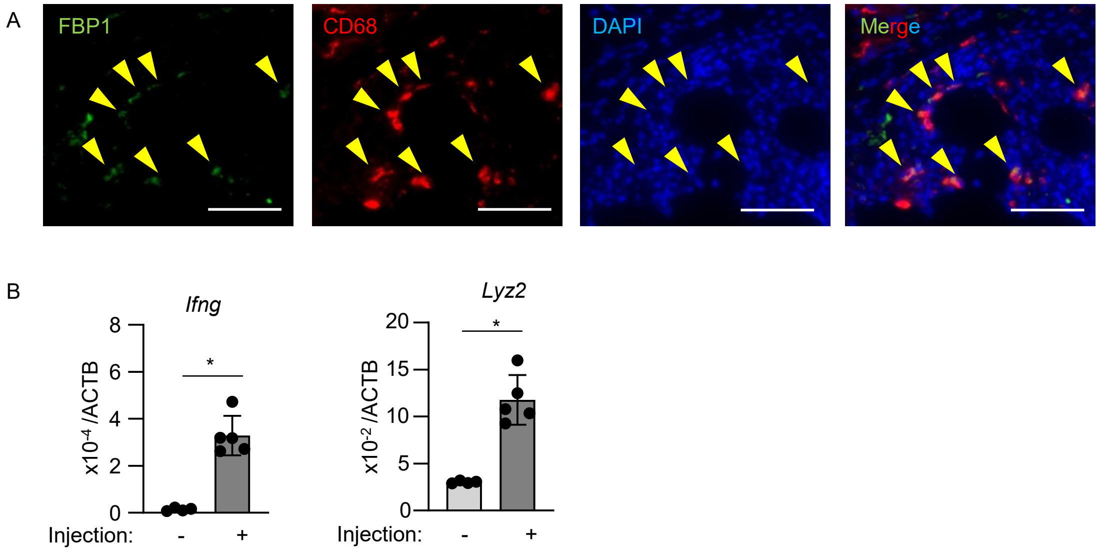

Supplementary Figure 7. Mouse granuloma model with subcutaneous injection of Con A and beads.

(**A**) Images of immunostained tissues three days after subcutaneous injection of Con A and beads to mouse skin (n=3). Green: FBP1, Red: CD68, Blue: DAPI. Scale bar = 50 µm. (**B**) Bar graph of quantitative PCR on mouse skin three days after subcutaneous injection of Con A and beads (n=5). Data are representative of three independent experiments.

Supplementary Table 1: Sample summary

Sample ID	Age	Sex	Body site	Disease
SS-1	47	F	Eyebrow	Sarcoidosis
SS-2	60	F	Left lower leg	Sarcoidosis
SS-3	31	M	Lower lip	Sarcoidosis
HS-1	85	F	Left cheek	Healthy
HS-2	52	F	Right lower leg	Healthy
HS-3	90	M	Right cheek	Healthy
HS-4	58	F	Face	Healthy
HS-5	63	M	Left upper arm	Healthy
